# Supplementary material for: Design, Synthesis, and In Vitro Evaluation of Benzofuro[3,2-c]Quinoline Derivatives as Potential Antileukemia Agents
Source: Molecules. 2020 Jan 3;25(1):203. doi: 10.3390/molecules25010203 (PMC6983037; doi:10.3390/molecules25010203)
Supplement: Supplementary file 1 [file molecules-25-00203-s001.pdf]

Supporting Information

Design, synthesis and in vitro evaluation of benzofuro[3,2-*c*]quinoline derivatives as potential antileukemia agents

Ying Lin[a], Dong Xing[a], Wen-Biao Wu[d,e], Gao-Ya Xu [d,e], Li-Fang Yu[a], Jie Tang[a,c], Yu-Bo Zhou[d,e], Jia Li [b,d,e*] and Fan Yang[a,*]

[a] Shanghai Engineering Research Center of Molecular Therapeutics and New Drug Development, School of Chemistry and Molecular Engineering, East China Normal University, Shanghai, 200062, PR China; fyang@chem.ecnu.edu.cn.

[b] Open Studio for Druggability Research of Marine Natural Products, Pilot National Laboratory for Marine Science and Technology (Qingdao), 1 Wenhai Road, Aoshanwei, Jimo, Qingdao, 266237, PR China.

[c] Shanghai Greenchem & Biotech Co., Ltd., Shanghai, 200062, PR China.

[d] National Center for Drug Screening, Shanghai Institute of Material Medica, Chinese Academy of Science, Shanghai, 201203, PR China; jli@simm.ac.cn.

[e] University of Chinese Academy of Sciences, No. 19A Yuquan Road, Beijing, 100049, PR China.

* Correspondence: fyang@chem.ecnu.edu.cn, jli@simm.ac.cn; Tel.:+86-21-62232764 (F.Y.), +86-21-50806600 (J. L.)

Received: date; Accepted: date; Published: date

1. Condition Optimization on the demethylation/cyclization

Table S1 Condition optimization on the demethyl-cyclization (one pot)

Entry	Condition	Tempo (℃)	Time (h)	Yield of **2a** (%)
1	48% HBr/AcOH	reflux	8	no product [1]
2	HI	reflux	12	no product [1]
3	BBr_3/DCM	reflux	8	no product [1]
4	Pyridine	reflux	5	no reaction [2]

[1] demethylation occurs smoothly while further cyclized **2a** could not obtained; [2] only starting material **1a**

Table S2 Condition optimization on the cyclization (stepwise) [1]

Entry	Condition	Tempo (℃)	Time (h)	Yield of **2a** (%)
1	AcOH/EtOH	reflux	6	no reaction
2	Pyridine	reflux	10	no reaction
3	KO*t*-Bu/*t*-BuOH	reflux	8	no reaction
4	NaH/DMF	100	7	no reaction

[1] demethylation of **1a** through BBr_3/DCM at room temperature, then next cyclization was optimized.

2. Condition Optimization on the cyclization

Table S3 Condition optimization on the cyclization from **4a** to **2a** [1]

Entry	Base	Solvent	Tempo (℃)	%Yield of **2a** (%) [2]
1	NaH	MeCN	70	82
2	KOH	MeCN	70	59
3	Cs_2CO_3	MeCN	70	83
4	KO*t*-Bu	MeCN	70	87
5	Et_3N	MeCN	70	n.r. [3]
6	Pyridine	MeCN	70	n.r.
7	KO*t*-Bu	CH_3CH_2OH	70	n.r.
8	KO*t*-Bu	THF	70	trace
9	KO*t*-Bu	*t*-BuOH	70	trace
10	KO*t*-Bu	DMF	70	89
11	KO*t*-Bu	DMF	60	88
12	KO*t*-Bu	DMF	50	70

[1] **4a** (1mmol), base (2 mmol), solvent (5 ml); [2] Isolated yield; [3] no reaction.

3. NMR charts

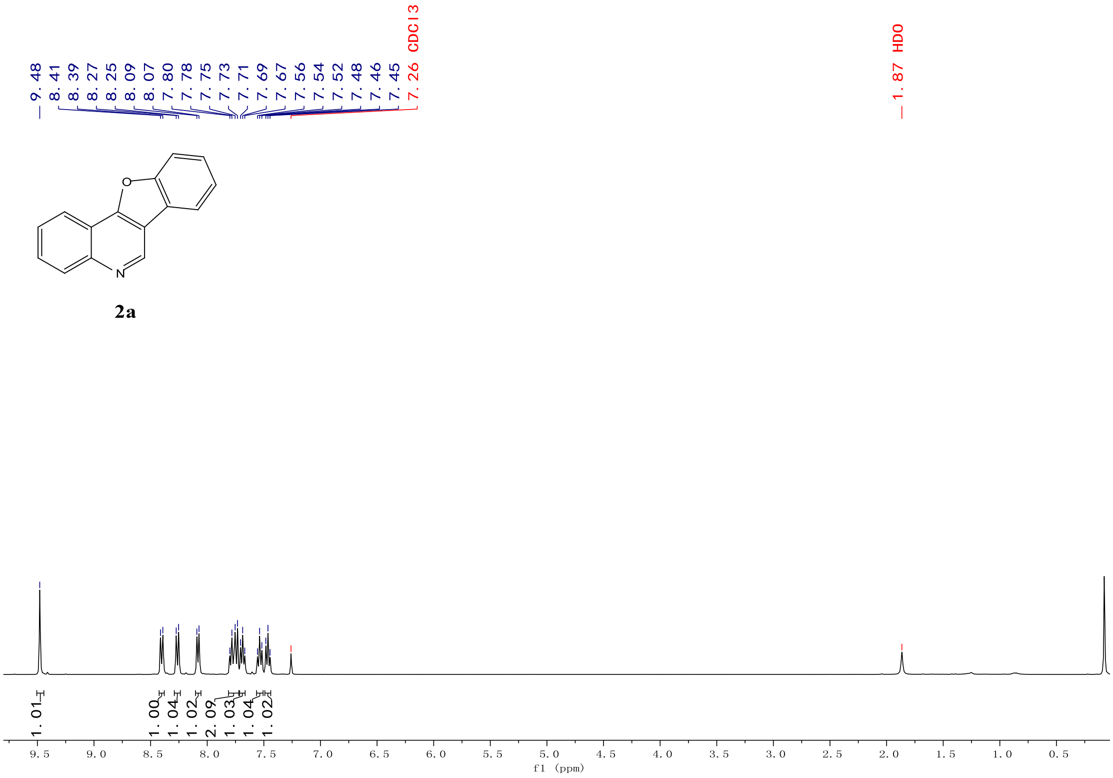

9.48
8.41
8.39
8.27
8.25
8.09
8.07
7.80
7.78
7.75
7.73
7.71
7.69
7.67
7.56
7.54
7.52
7.48
7.46
7.45
7.26 CDCl3
1.87 HDO
O
N
2a
1.01
1.00
1.04
1.02
2.09
1.03
1.04
1.02
9.5 9.0 8.5 8.0 7.5 7.0 6.5 6.0 5.5 5.0 4.5 4.0 3.5 3.0 2.5 2.0 1.5 1.0 0.5
f1 (ppm)

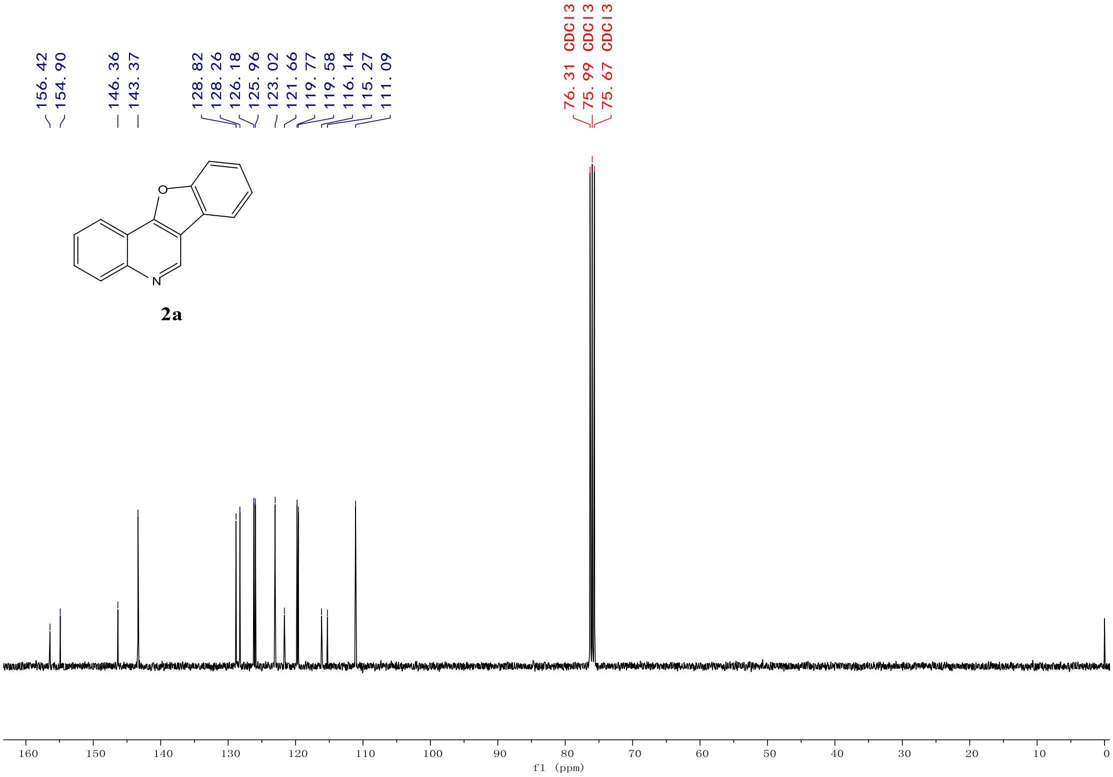

156.42
154.90
146.36
143.37
128.82
128.26
126.18
125.96
123.02
121.66
119.77
119.58
116.14
115.27
111.09
76.31 CDCl3
75.99 CDCl3
75.67 CDCl3
O
N
2a
160 150 140 130 120 110 100 90 80 70 60 50 40 30 20 10 0
f1 (ppm)

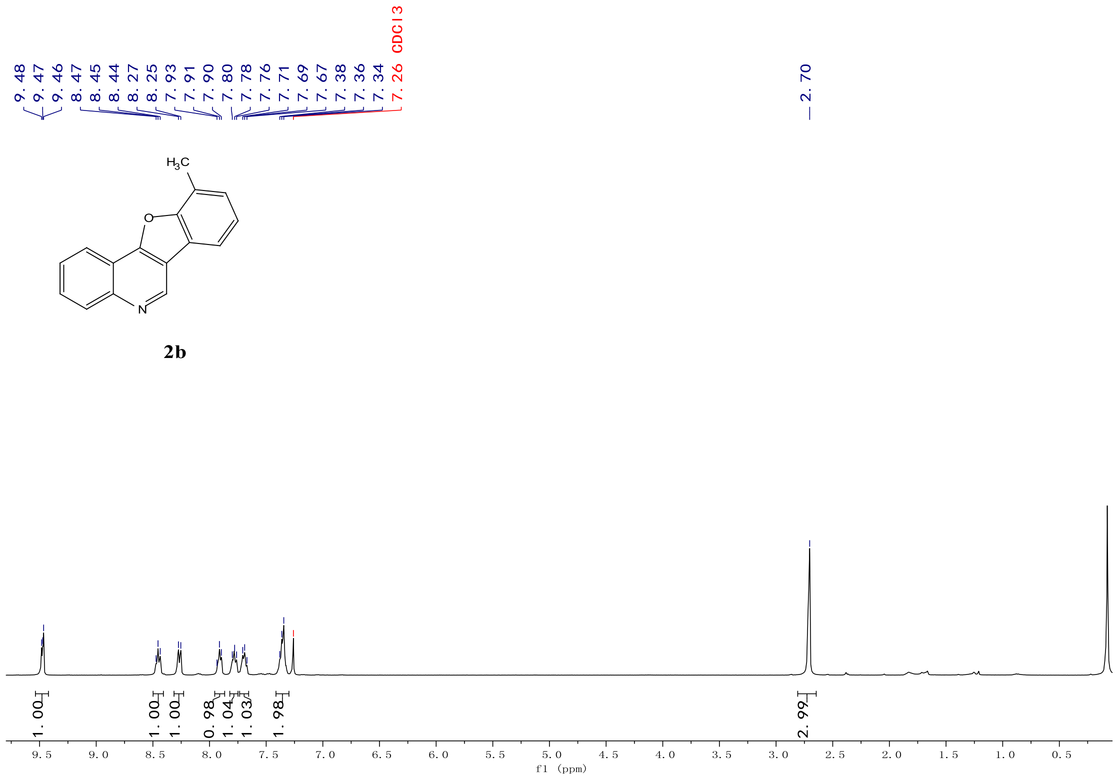
9.48
9.47
9.46
8.47
8.45
8.44
8.27
8.25
7.93
7.91
7.90
7.80
7.78
7.76
7.71
7.69
7.67
7.38
7.36
7.34
7.26 CDCl3
2.70
H3C
O
N
2b
1.00
1.00
1.00
0.98
1.04
1.03
1.98
2.99
f1 (ppm)

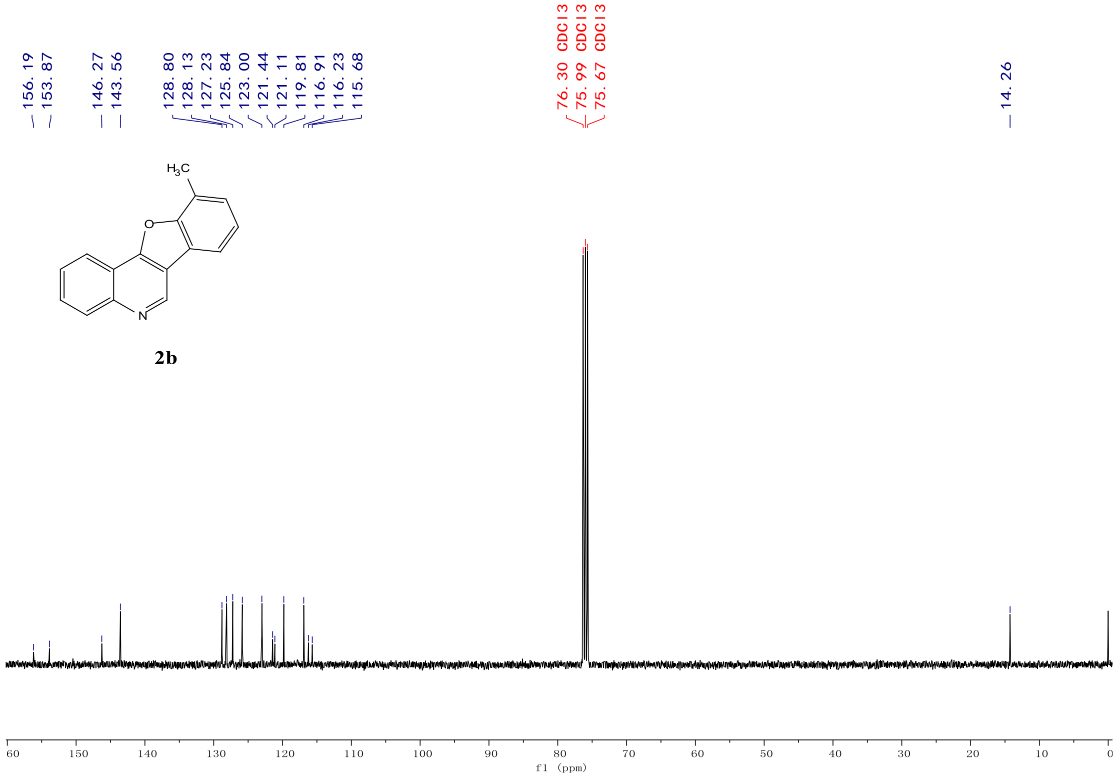
156.19
153.87
146.27
143.56
128.80
128.13
127.23
125.84
123.00
121.44
121.11
119.81
116.91
116.23
115.68
76.30 CDCl3
75.99 CDCl3
75.67 CDCl3
14.26
H3C
O
N
2b
f1 (ppm)

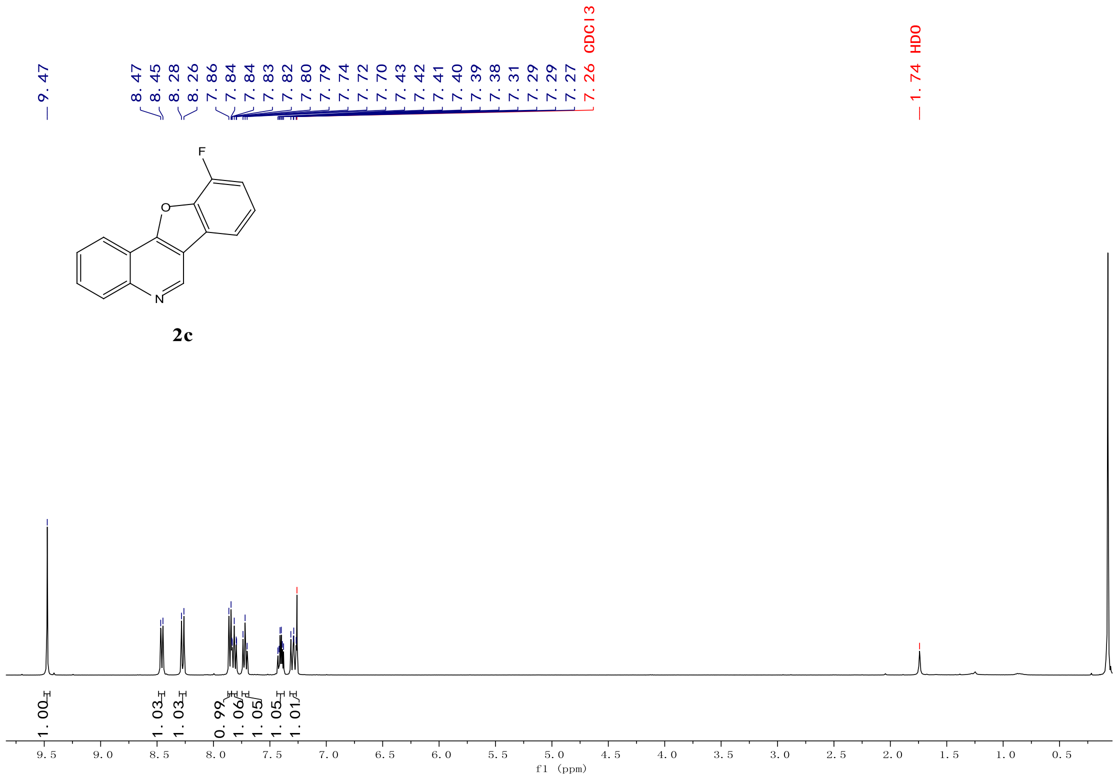
9.47
8.47
8.45
8.28
8.26
7.86
7.84
7.84
7.83
7.82
7.80
7.79
7.74
7.72
7.70
7.43
7.42
7.41
7.40
7.39
7.38
7.31
7.29
7.27
7.26 CDCl3
1.74 HDO
F
O
N
2c
1.00
1.03
1.03
0.99
1.06
1.05
1.05
1.01
f1 (ppm)

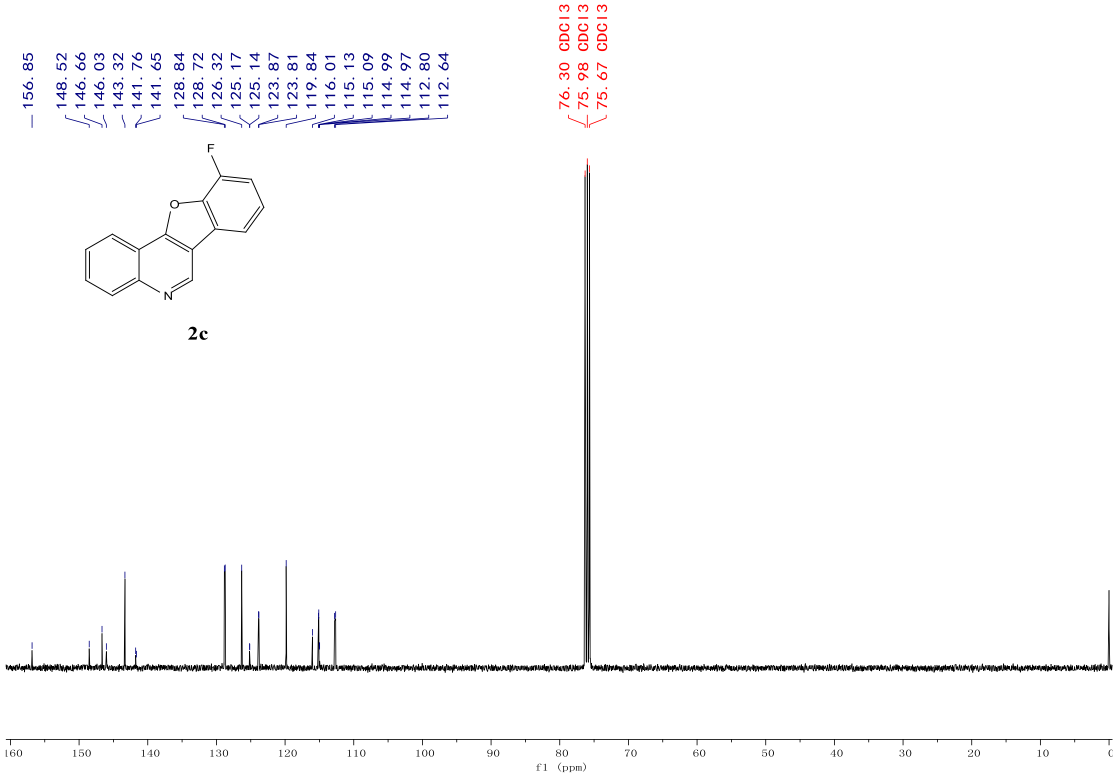
156.85
148.52
146.66
146.03
143.32
141.76
141.65
128.84
128.72
126.32
125.17
125.14
123.87
123.81
119.84
116.01
115.13
115.09
114.99
114.97
112.80
112.64
76.30 CDCl3
75.98 CDCl3
75.67 CDCl3
F
O
N
2c
f1 (ppm)

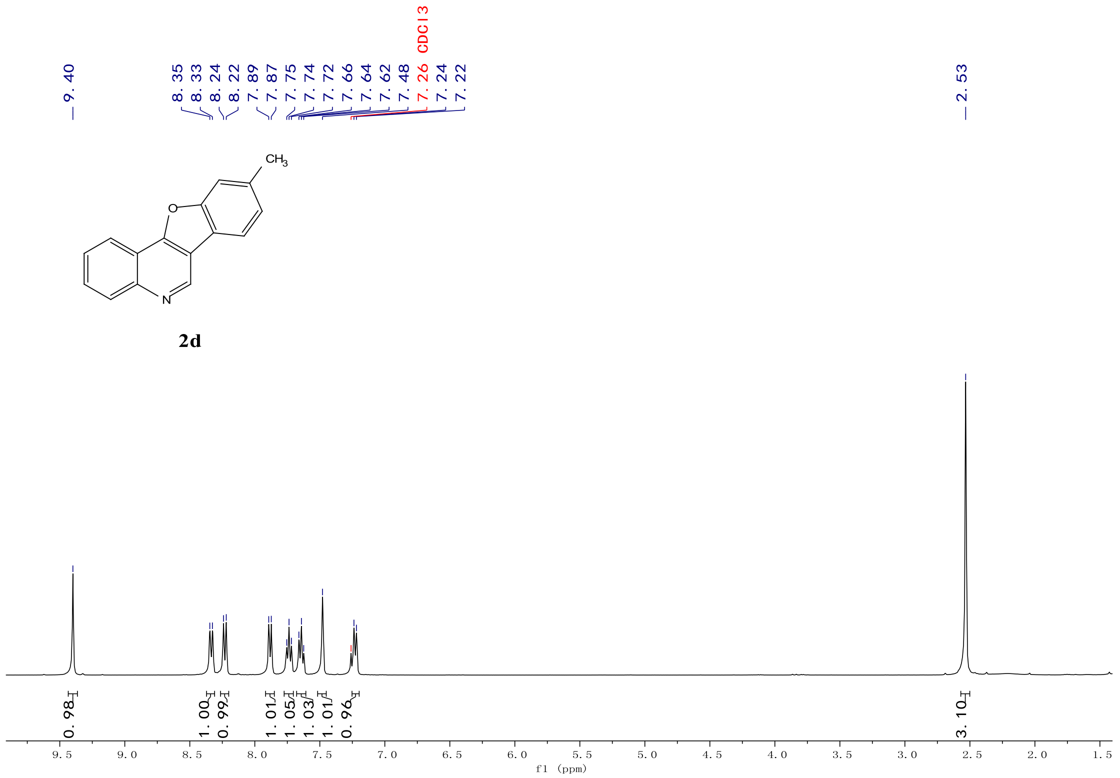
9.40
8.35
8.33
8.24
8.22
7.89
7.87
7.75
7.74
7.72
7.66
7.64
7.62
7.48
7.26 CDCl3
7.24
7.22
2.53
CH3
O
N
2d
0.98
1.00
0.99
1.01
1.05
1.03
1.01
0.96
3.10
f1 (ppm)

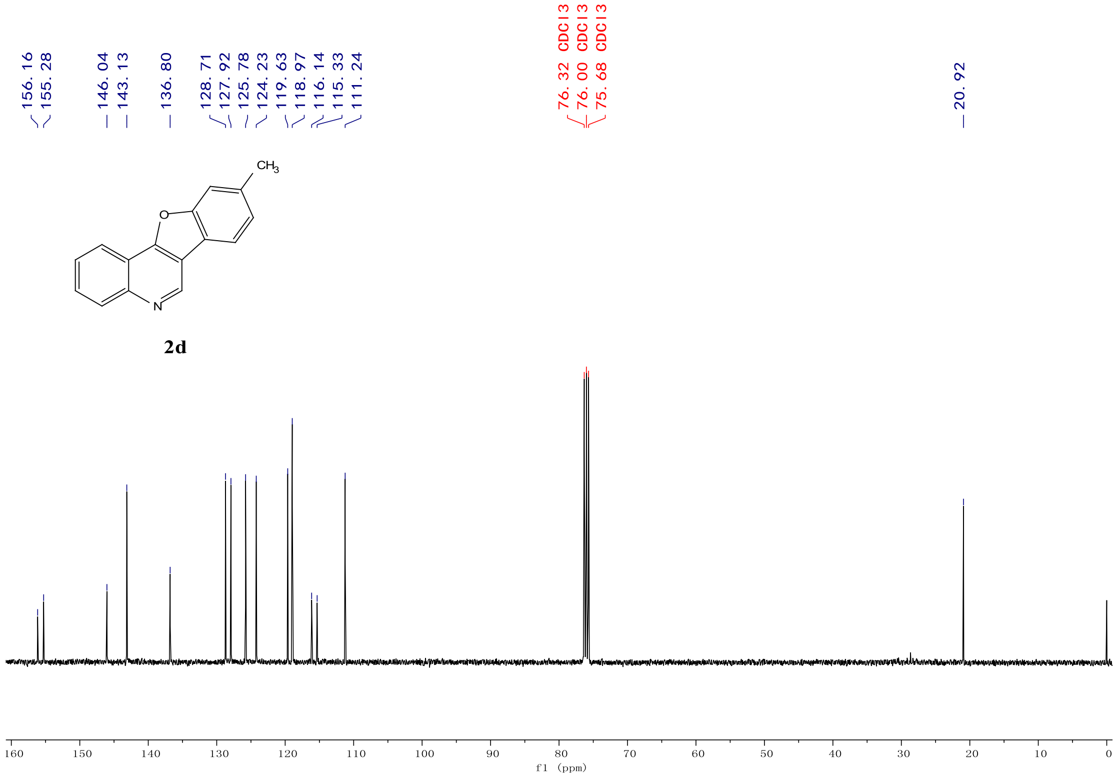
156.16
155.28
146.04
143.13
136.80
128.71
127.92
125.78
124.23
119.63
118.97
116.14
115.33
111.24
76.32 CDCl3
76.00 CDCl3
75.68 CDCl3
20.92
CH3
O
N
2d
f1 (ppm)

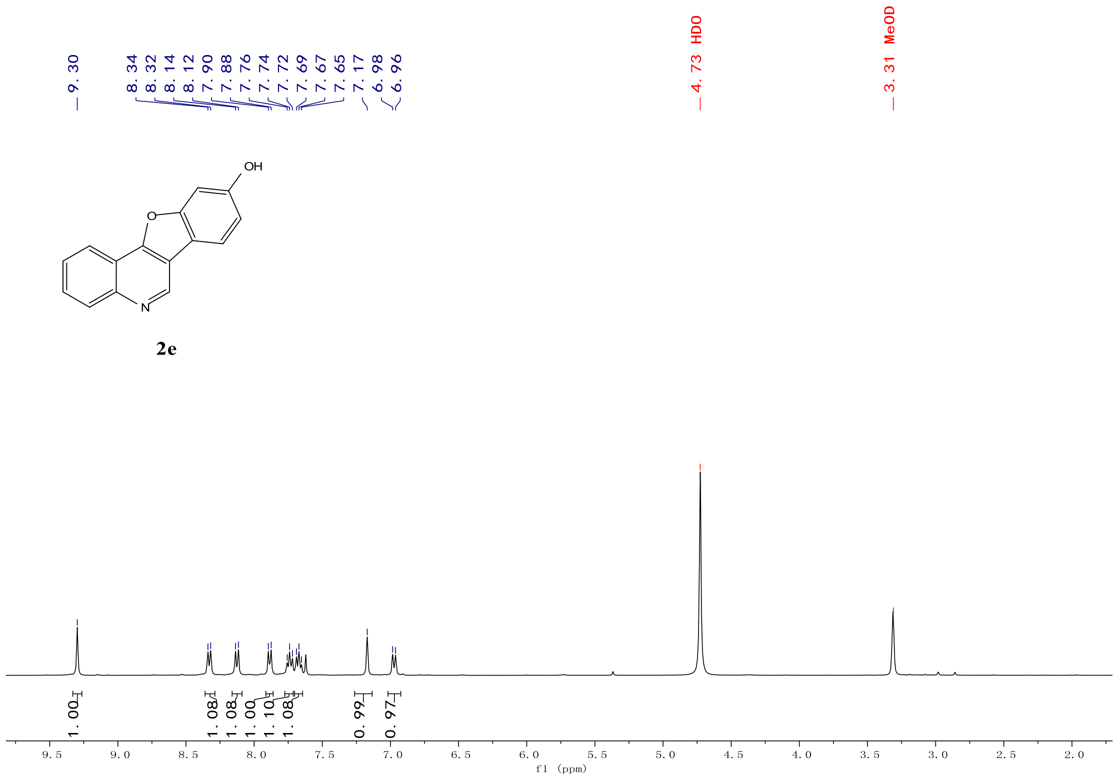
9.30
8.34
8.32
8.14
8.12
7.90
7.88
7.76
7.74
7.72
7.69
7.67
7.65
7.17
6.98
6.96
4.73 HDO
3.31 MeOD
OH
O
N
2e
1.00
1.08
1.08
1.00
1.10
1.08
0.99
0.97
f1 (ppm)

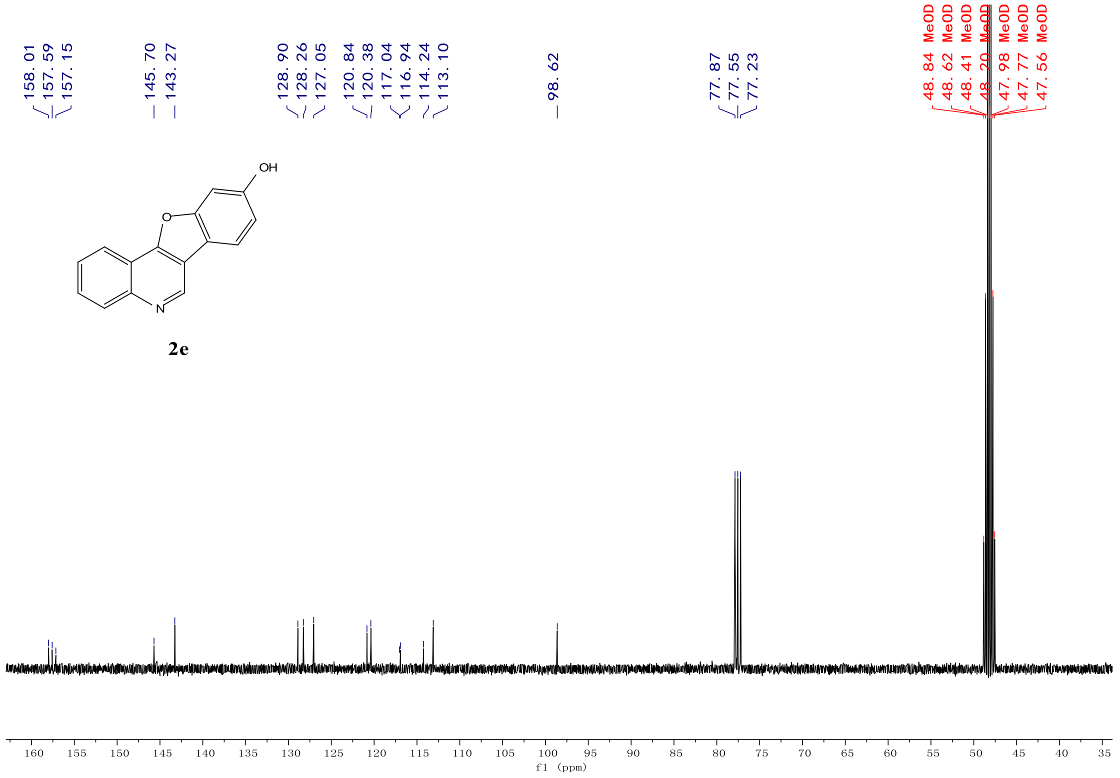
158.01
157.59
157.15
145.70
143.27
128.90
128.26
127.05
120.84
120.38
117.04
116.94
114.24
113.10
98.62
77.87
77.55
77.23
48.84 MeOD
48.62 MeOD
48.41 MeOD
48.20 MeOD
47.98 MeOD
47.77 MeOD
47.56 MeOD
OH
O
N
2e
f1 (ppm)

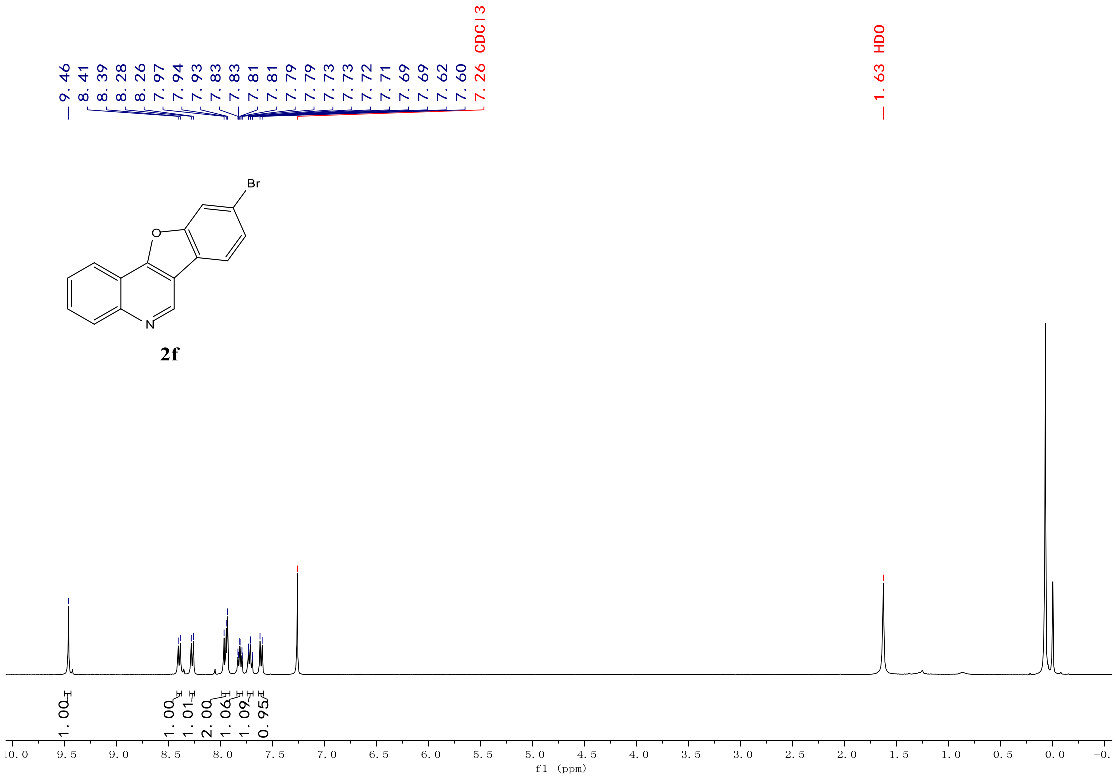
9.46
8.41
8.39
8.28
8.26
7.97
7.94
7.93
7.83
7.83
7.81
7.81
7.79
7.79
7.73
7.73
7.72
7.71
7.69
7.69
7.62
7.60
7.26 CDCl3
1.63 HDO
Br
O
N
2f
1.00
1.00
1.01
2.00
1.06
1.09
0.95
f1 (ppm)

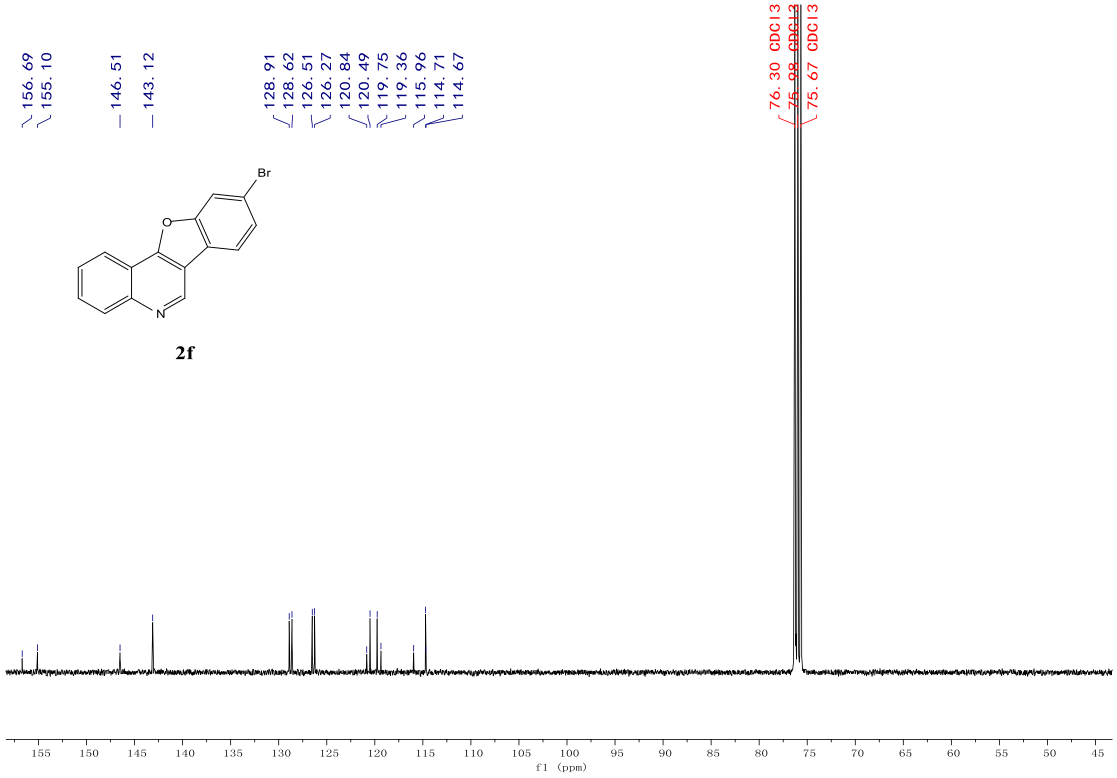
156.69
155.10
146.51
143.12
128.91
128.62
126.51
126.27
120.84
120.49
119.75
119.36
115.96
114.71
114.67
76.30 CDCl3
75.98 CDCl3
75.67 CDCl3
Br
O
N
2f
f1 (ppm)

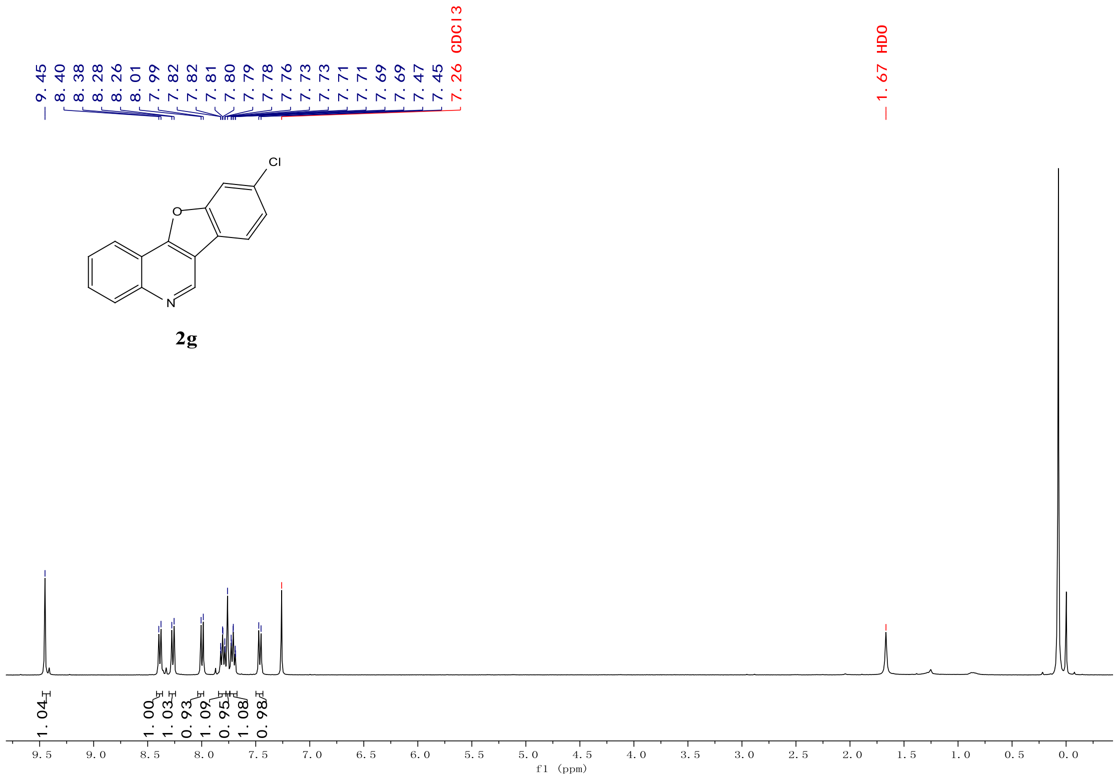
9.45
8.40
8.38
8.28
8.26
8.01
7.99
7.82
7.82
7.81
7.80
7.79
7.78
7.76
7.73
7.73
7.71
7.71
7.69
7.69
7.47
7.45
7.26 CDCl3
1.67 HDO
Cl
O
N
2g
1.04
1.00
1.03
0.93
1.09
0.95
1.08
0.98
f1 (ppm)

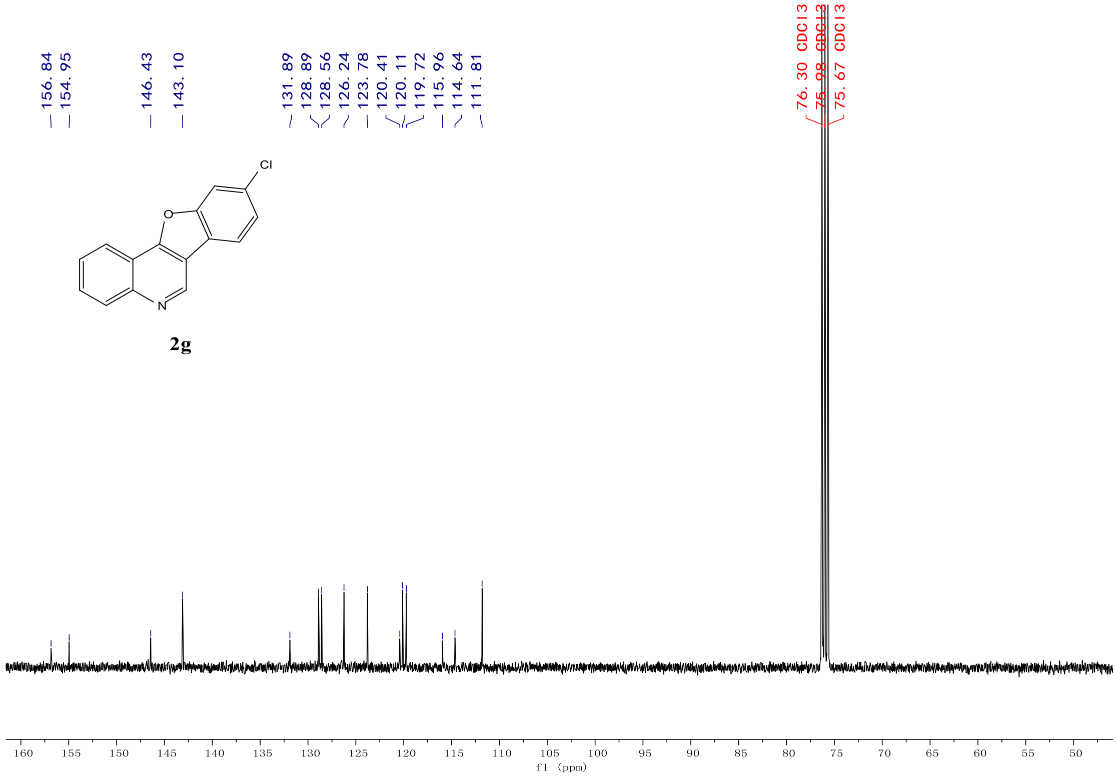
156.84
154.95
146.43
143.10
131.89
128.89
128.56
126.24
123.78
120.41
120.11
119.72
115.96
114.64
111.81
76.30 CDCl3
75.98 CDCl3
75.67 CDCl3
Cl
O
N
2g
f1 (ppm)

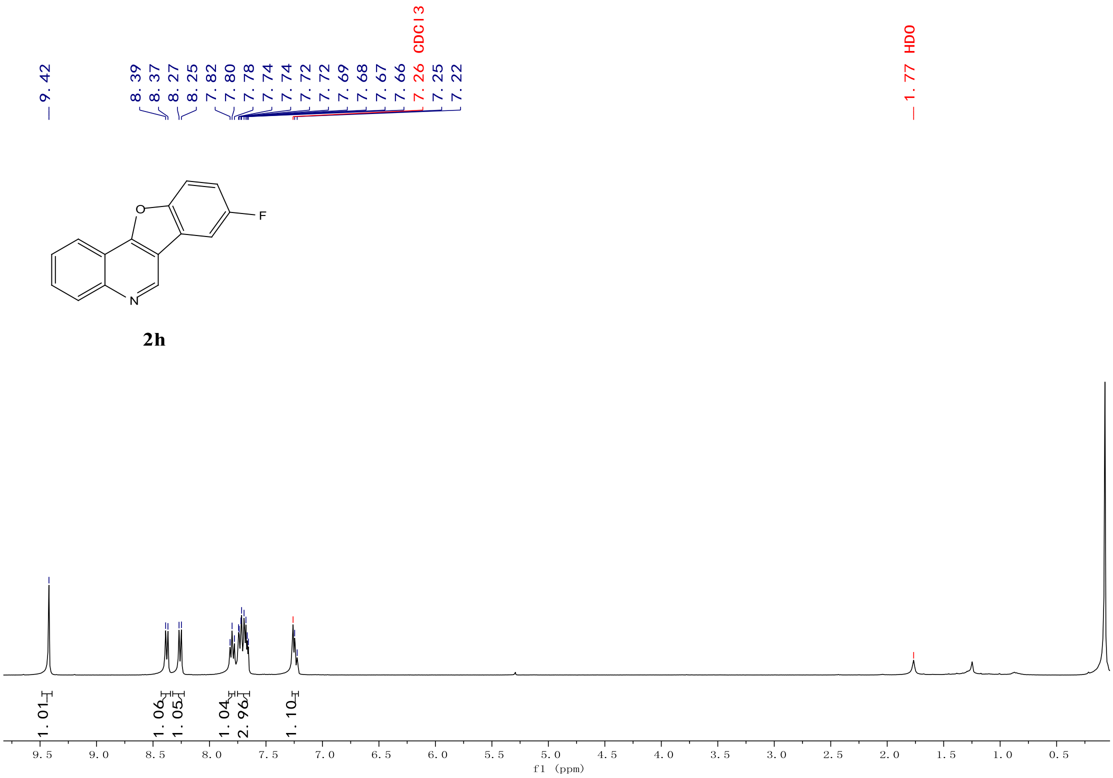
9.42
8.39 8.37 8.27 8.25 7.82 7.80 7.78 7.74 7.74 7.72 7.72 7.69 7.68 7.67 7.66 7.26 CDCl3 7.25 7.22
1.77 HDO
O
F
N
2h
1.01 1.06 1.05 1.04 2.96 1.10
9.5 9.0 8.5 8.0 7.5 7.0 6.5 6.0 5.5 5.0 4.5 4.0 3.5 3.0 2.5 2.0 1.5 1.0 0.5
f1 (ppm)

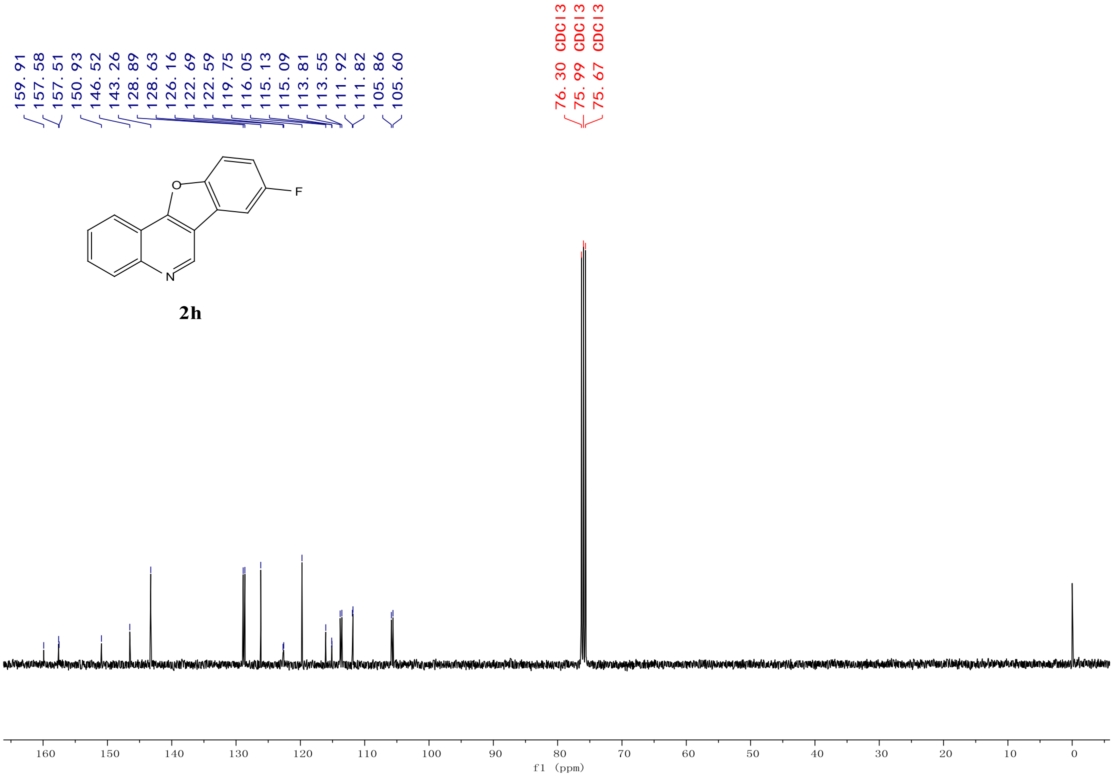
159.91 157.58 157.51 150.93 146.52 143.26 128.89 128.63 126.16 122.69 122.59 119.75 116.05 115.13 115.09 113.81 113.55 111.92 111.82 105.86 105.60
76.30 CDCl3 75.99 CDCl3 75.67 CDCl3
O
F
N
2h
160 150 140 130 120 110 100 90 80 70 60 50 40 30 20 10 0
f1 (ppm)

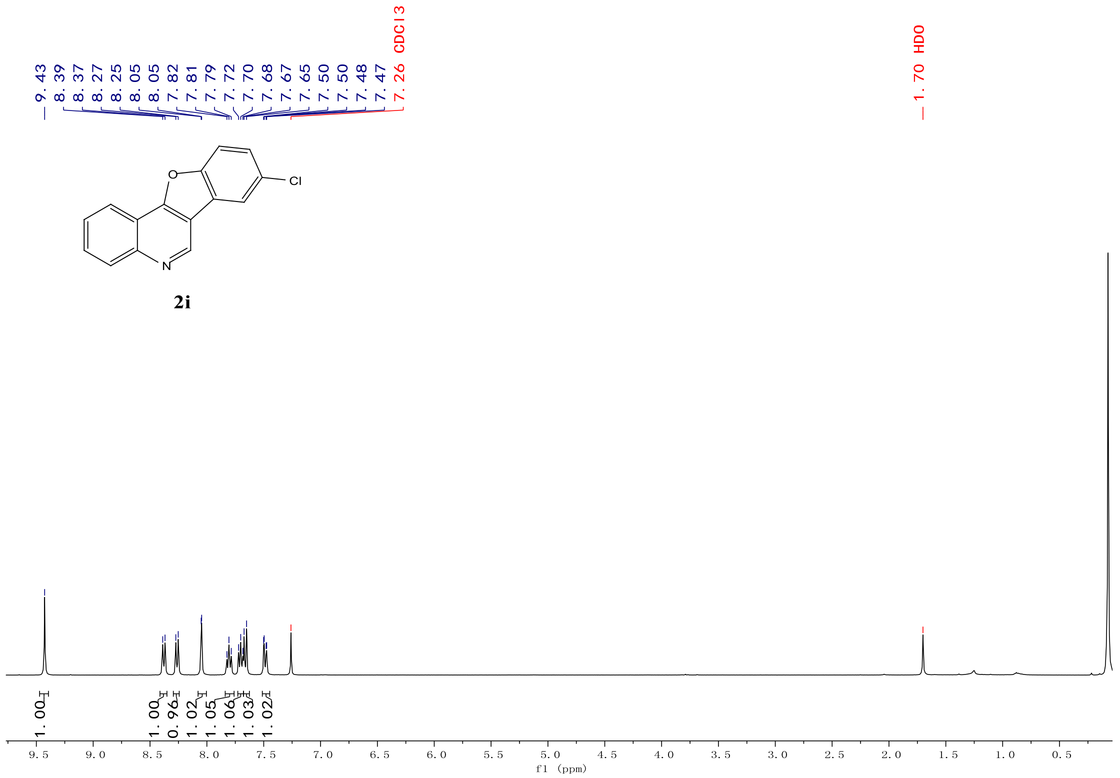
9.43
8.39
8.37
8.27
8.25
8.05
8.05
7.82
7.81
7.79
7.72
7.70
7.68
7.67
7.65
7.50
7.50
7.48
7.47
7.26 CDCl3
1.70 HDO
Cl
O
N
2i
1.00
1.00
0.96
1.02
1.05
1.06
1.03
1.02
f1 (ppm)

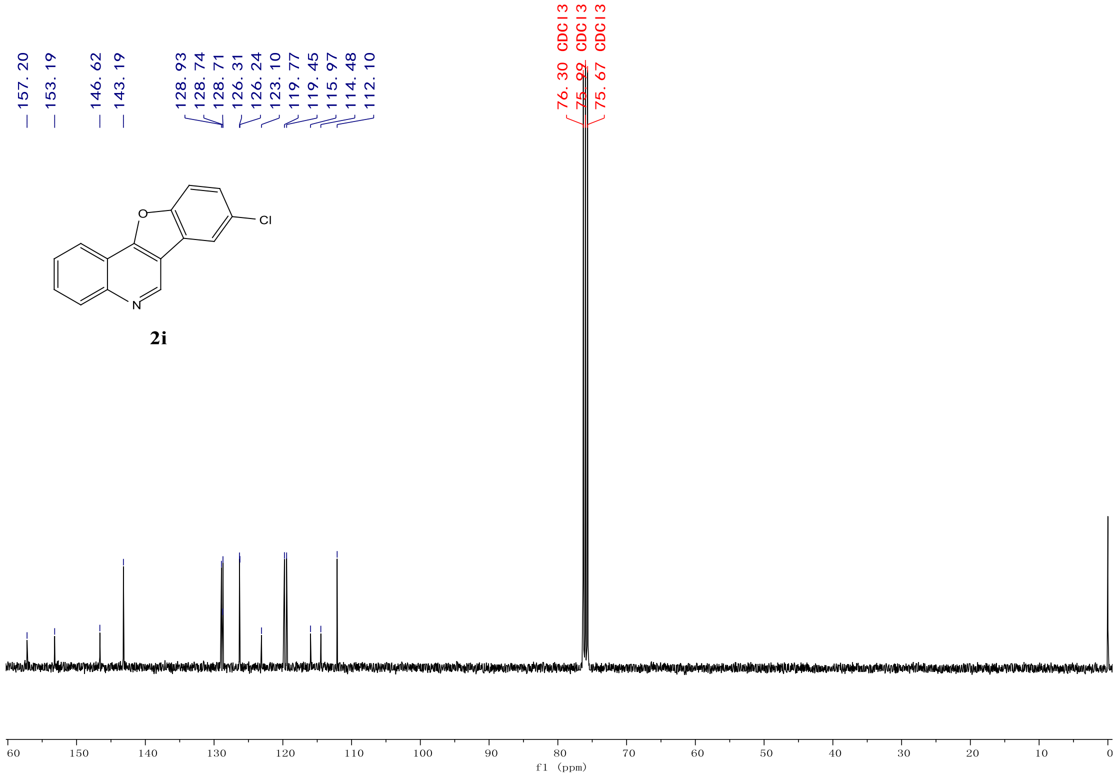
157.20
153.19
146.62
143.19
128.93
128.74
128.71
126.31
126.24
123.10
119.77
119.45
115.97
114.48
112.10
76.30 CDCl3
75.99 CDCl3
75.67 CDCl3
Cl
O
N
2i
f1 (ppm)

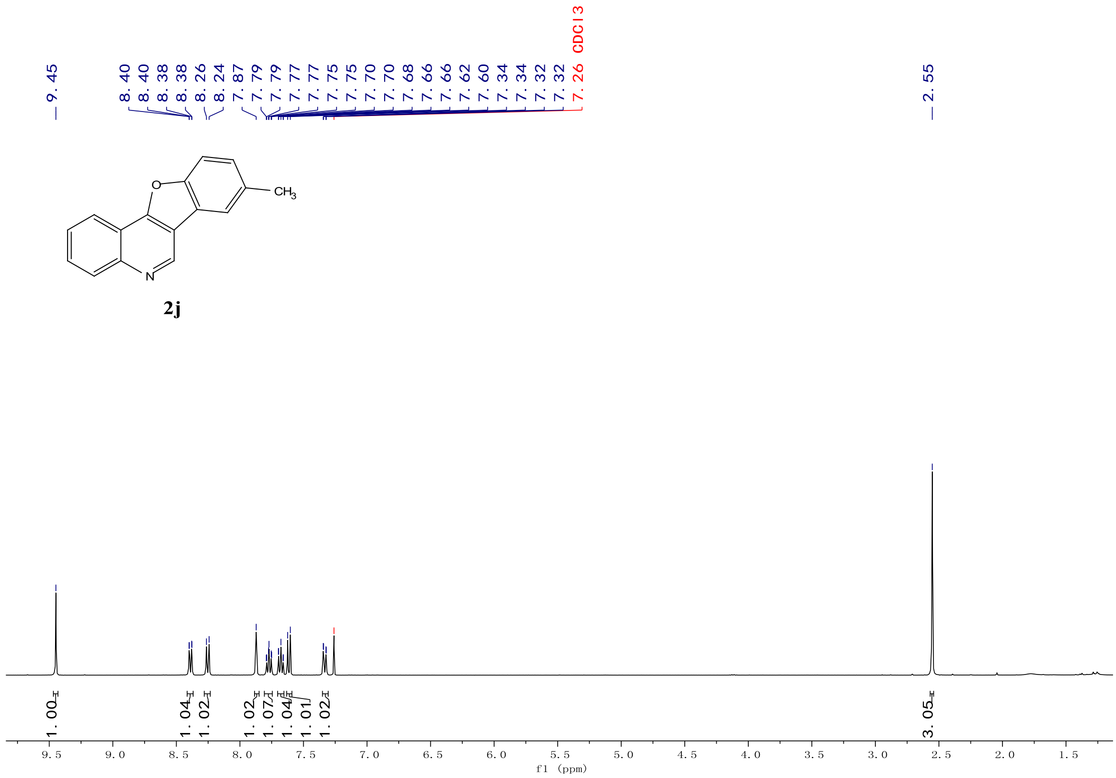
CDCl3
9.45
8.40
8.40
8.38
8.38
8.26
8.24
7.87
7.79
7.79
7.77
7.77
7.75
7.75
7.70
7.70
7.68
7.66
7.66
7.62
7.60
7.34
7.34
7.32
7.32
7.26
2.55
CH3
O
N
2j
1.00
1.04
1.02
1.02
1.07
1.04
1.01
1.02
3.05
f1 (ppm)

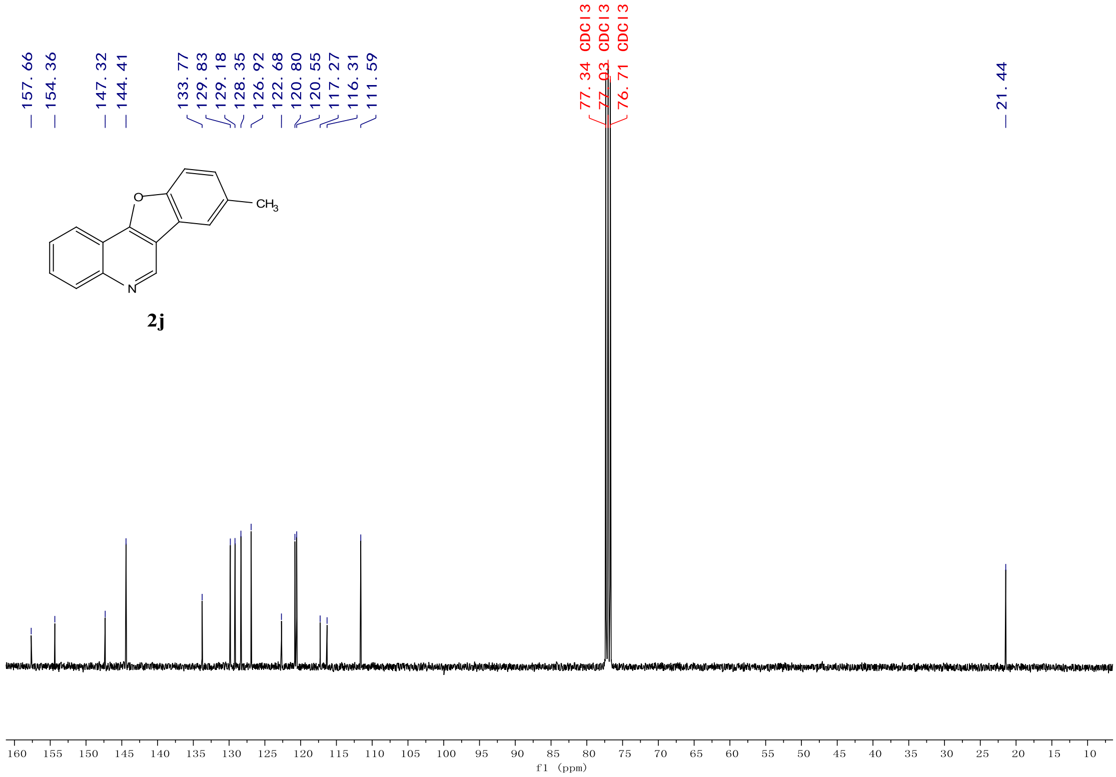
157.66
154.36
147.32
144.41
133.77
129.83
129.18
128.35
126.92
122.68
120.80
120.55
117.27
116.31
111.59
77.34 CDCl3
77.03 CDCl3
76.71 CDCl3
21.44
CH3
O
N
2j
f1 (ppm)

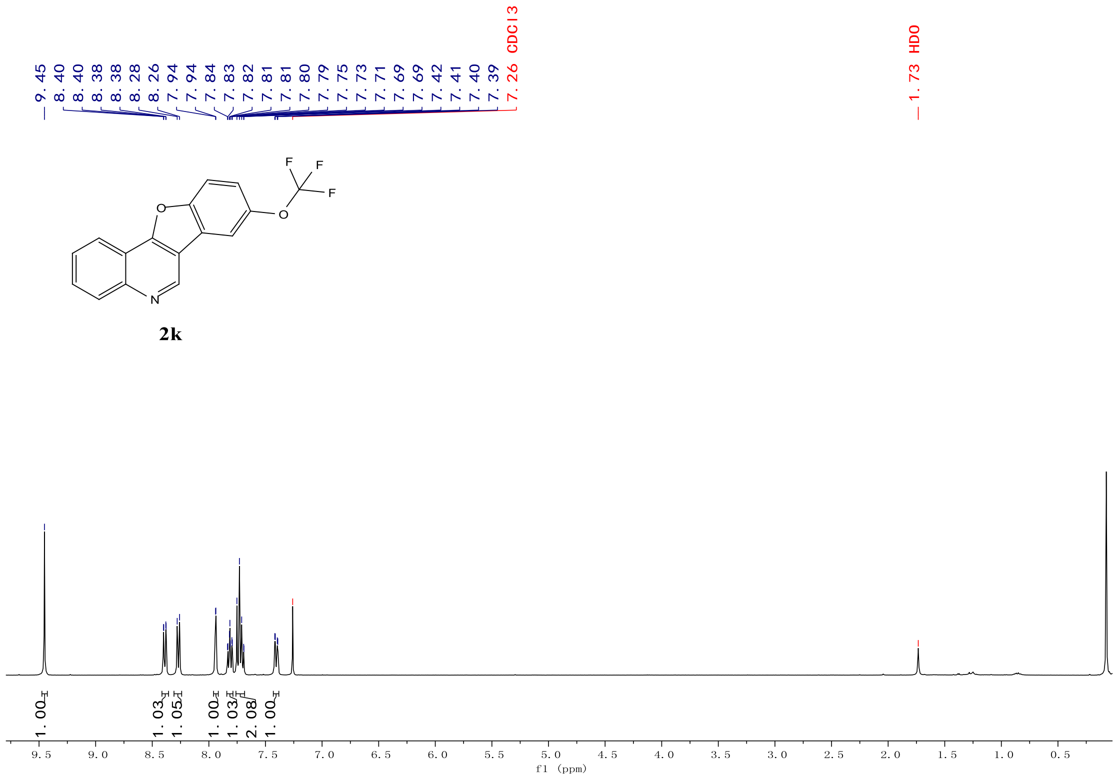
9.45
8.40
8.38
8.38
8.28
8.26
7.94
7.94
7.84
7.83
7.82
7.81
7.81
7.80
7.79
7.75
7.73
7.71
7.69
7.42
7.41
7.40
7.39
7.26 CDCl3
1.73 HDO
2k
1.00
1.03
1.05
1.00
1.03
2.08
1.00
f1 (ppm)

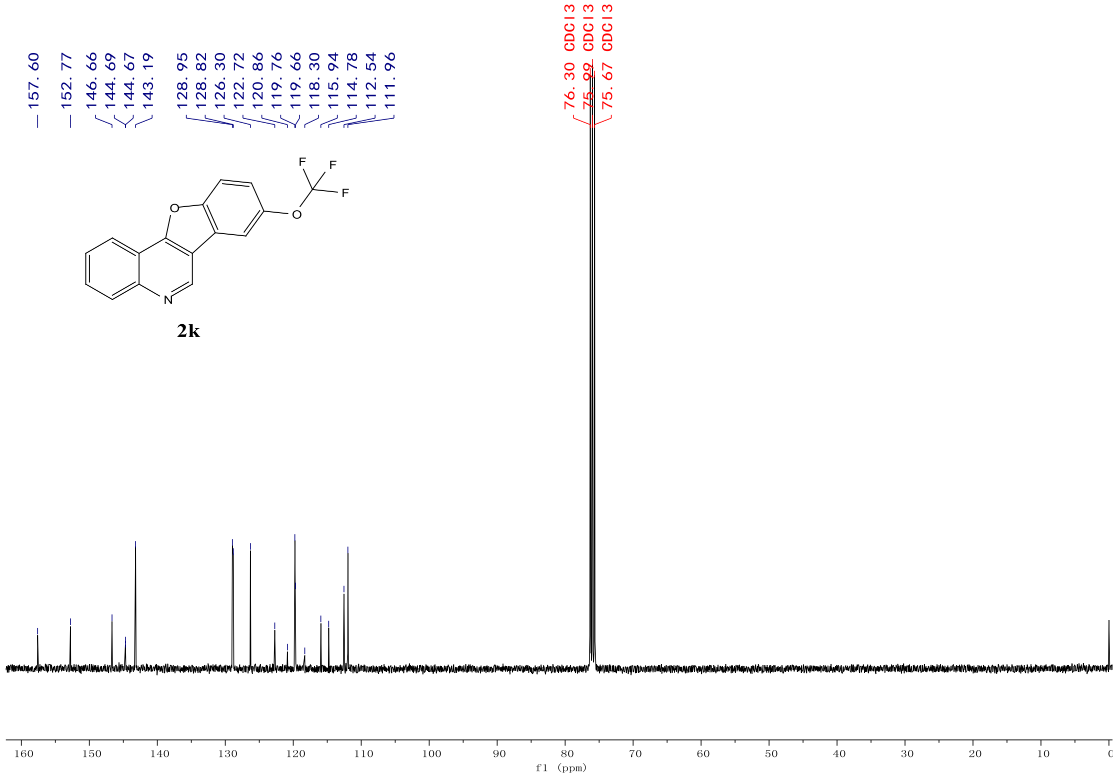
157.60
152.77
146.66
144.69
144.67
143.19
128.95
128.82
126.30
122.72
120.86
119.76
119.66
118.30
115.94
114.78
112.54
111.96
76.30 CDCl3
75.99 CDCl3
75.67 CDCl3
2k
f1 (ppm)

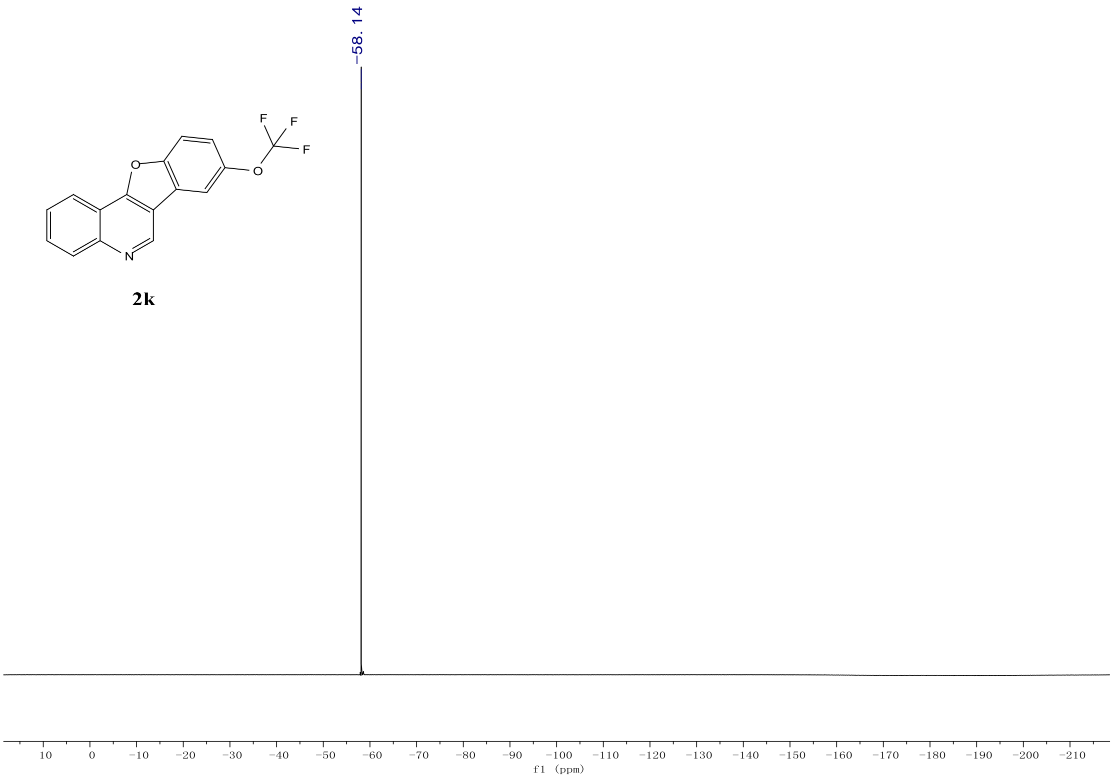

−58.14
2k
10 0 −10 −20 −30 −40 −50 −60 −70 −80 −90 −100 −110 −120 −130 −140 −150 −160 −170 −180 −190 −200 −210
f1 (ppm)

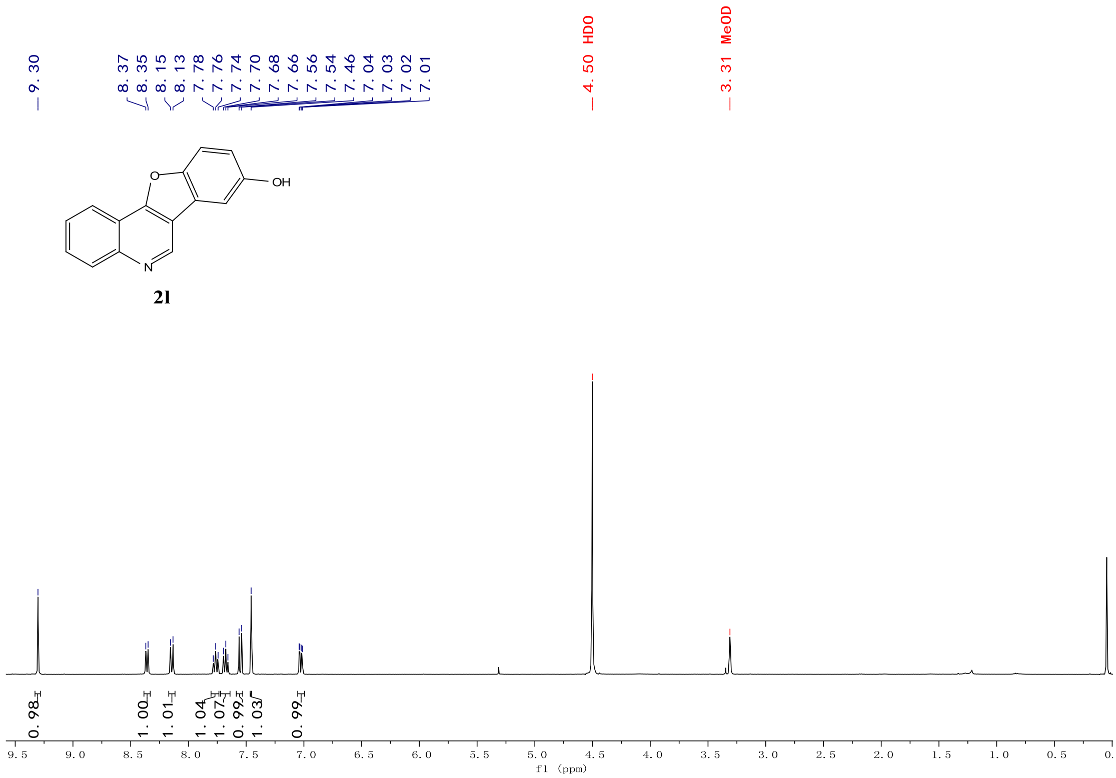
9.30
8.37
8.35
8.15
8.13
7.78
7.76
7.74
7.70
7.68
7.66
7.56
7.54
7.46
7.04
7.03
7.02
7.01
4.50 HDO
3.31 MeOD
OH
O
N
2l
0.98
1.00
1.01
1.04
1.07
0.99
1.03
0.99
f1 (ppm)

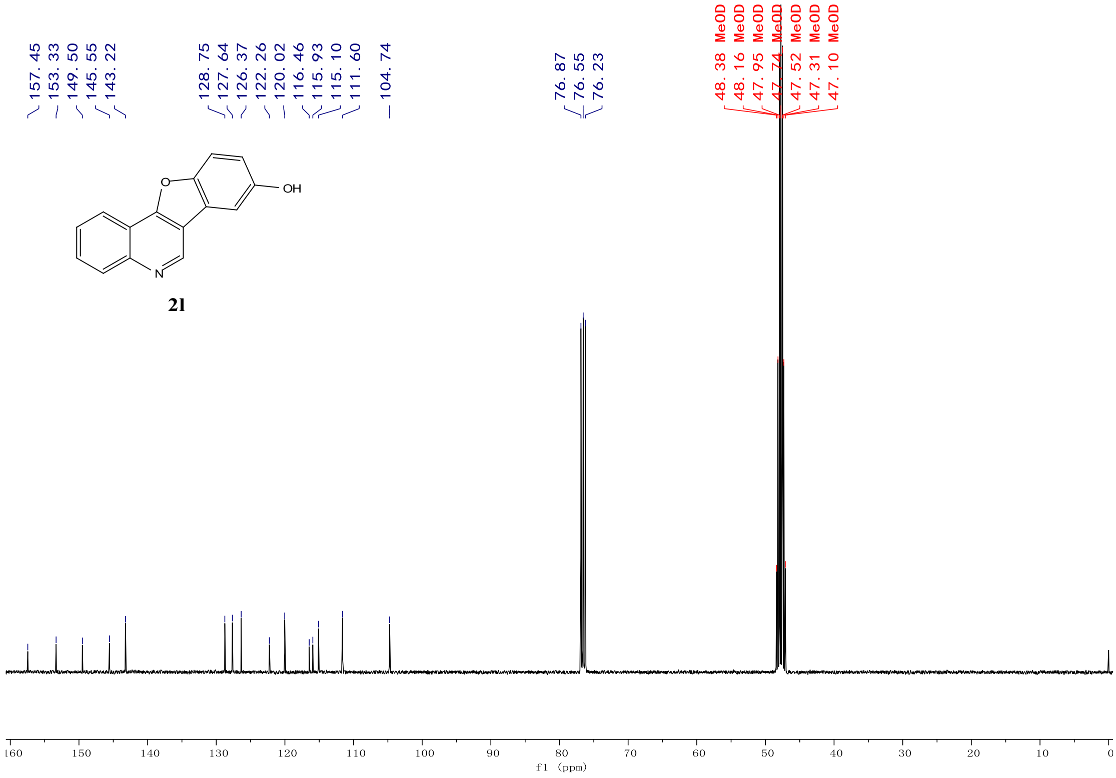
157.45
153.33
149.50
145.55
143.22
128.75
127.64
126.37
122.26
120.02
116.46
115.93
115.10
111.60
104.74
76.87
76.55
76.23
48.38 MeOD
48.16 MeOD
47.95 MeOD
47.74 MeOD
47.52 MeOD
47.31 MeOD
47.10 MeOD
OH
O
N
2l
f1 (ppm)

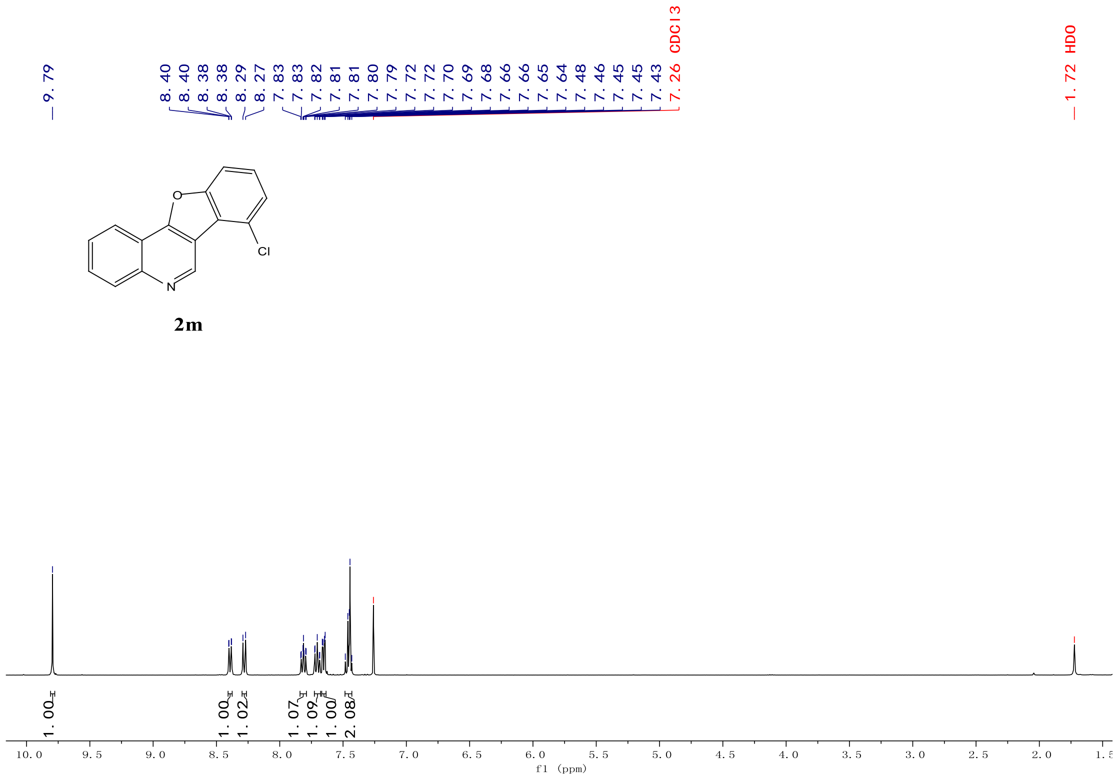
9.79
8.40
8.40
8.38
8.38
8.29
8.27
7.83
7.82
7.81
7.81
7.80
7.79
7.72
7.72
7.70
7.69
7.68
7.66
7.66
7.65
7.64
7.48
7.46
7.45
7.45
7.43
7.26 CDCl3
1.72 HDO
O
Cl
N
2m
1.00
1.00
1.02
1.07
1.09
1.00
2.08
10.0 9.5 9.0 8.5 8.0 7.5 7.0 6.5 6.0 5.5 5.0 4.5 4.0 3.5 3.0 2.5 2.0 1.5
f1 (ppm)

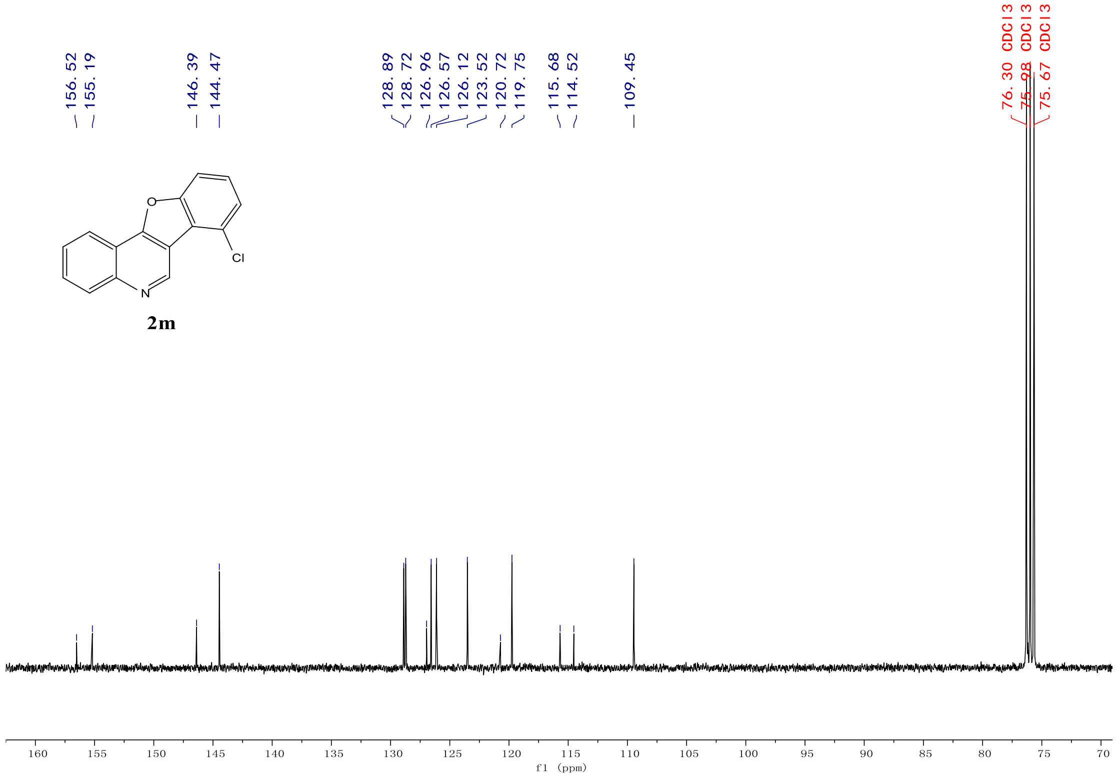
156.52
155.19
146.39
144.47
128.89
128.72
126.96
126.57
126.12
123.52
120.72
119.75
115.68
114.52
109.45
76.30 CDCl3
75.98 CDCl3
75.67 CDCl3
O
Cl
N
2m
160 155 150 145 140 135 130 125 120 115 110 105 100 95 90 85 80 75 70
f1 (ppm)

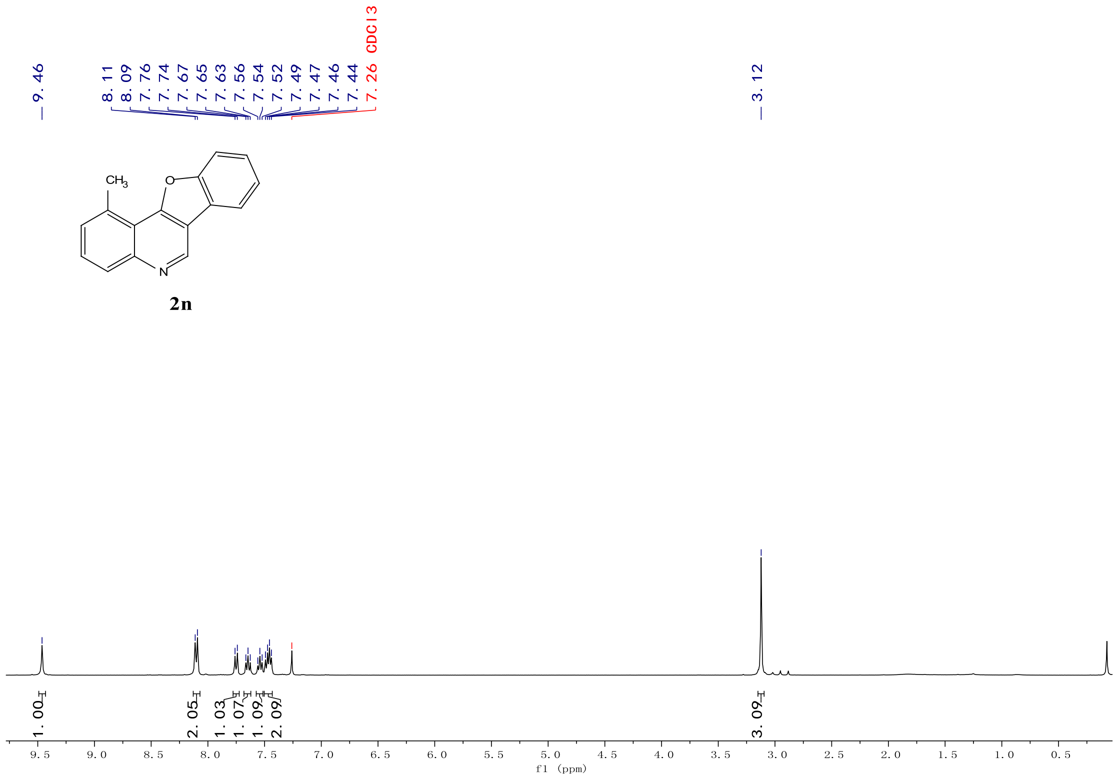
9.46
8.11
8.09
7.76
7.74
7.67
7.65
7.63
7.56
7.54
7.52
7.49
7.47
7.46
7.44
7.26 CDCl3
3.12
CH3
O
N
2n
1.00
2.05
1.03
1.07
1.09
2.09
3.09
9.5 9.0 8.5 8.0 7.5 7.0 6.5 6.0 5.5 5.0 4.5 4.0 3.5 3.0 2.5 2.0 1.5 1.0 0.5
f1 (ppm)

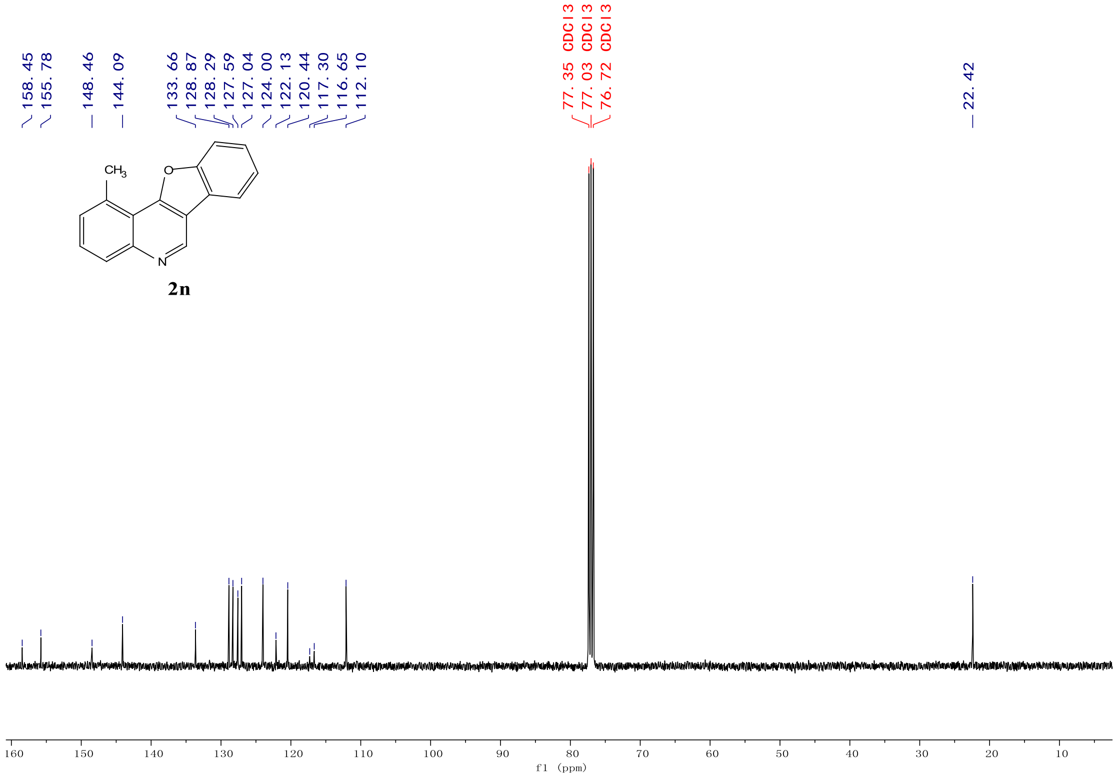
158.45
155.78
148.46
144.09
133.66
128.87
128.29
127.59
127.04
124.00
122.13
120.44
117.30
116.65
112.10
77.35 CDCl3
77.03 CDCl3
76.72 CDCl3
22.42
CH3
O
N
2n
160 150 140 130 120 110 100 90 80 70 60 50 40 30 20 10
f1 (ppm)

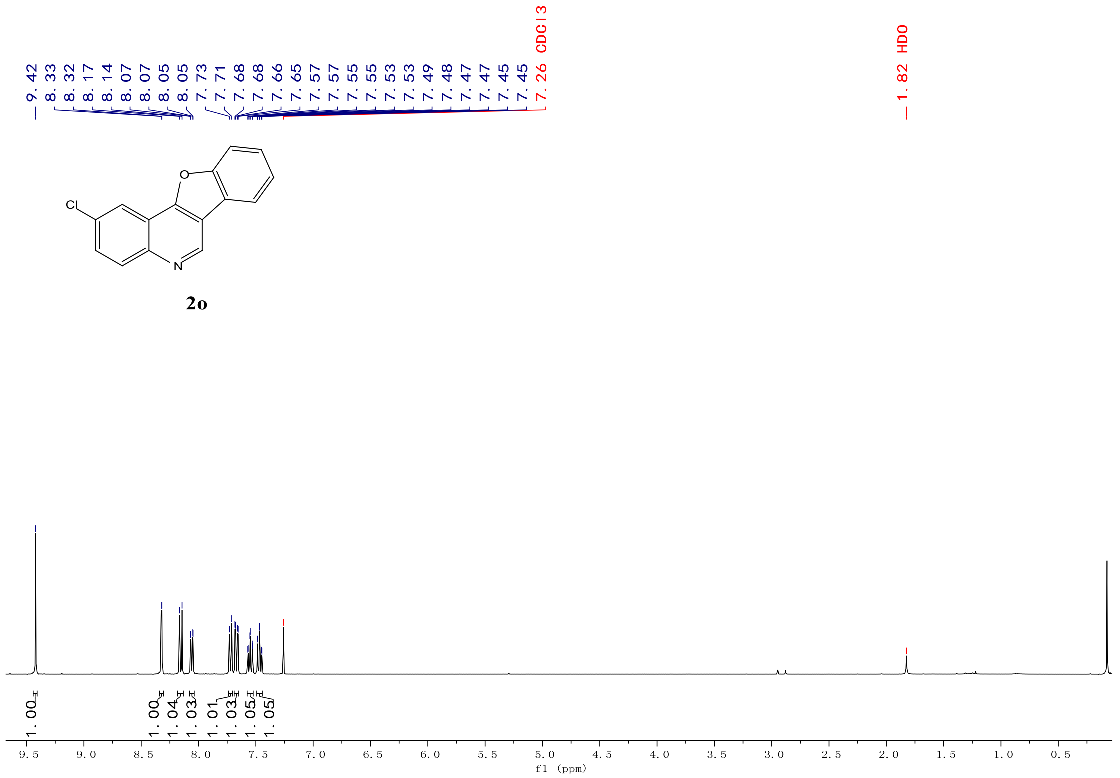
9.42
8.33
8.32
8.17
8.14
8.07
8.07
8.05
8.05
7.73
7.71
7.68
7.68
7.66
7.65
7.57
7.57
7.55
7.55
7.53
7.53
7.49
7.48
7.47
7.47
7.45
7.45
7.26 CDCl3
1.82 HDO
Cl
O
N
2o
1.00
1.00
1.04
1.03
1.01
1.03
1.05
1.05
f1 (ppm)

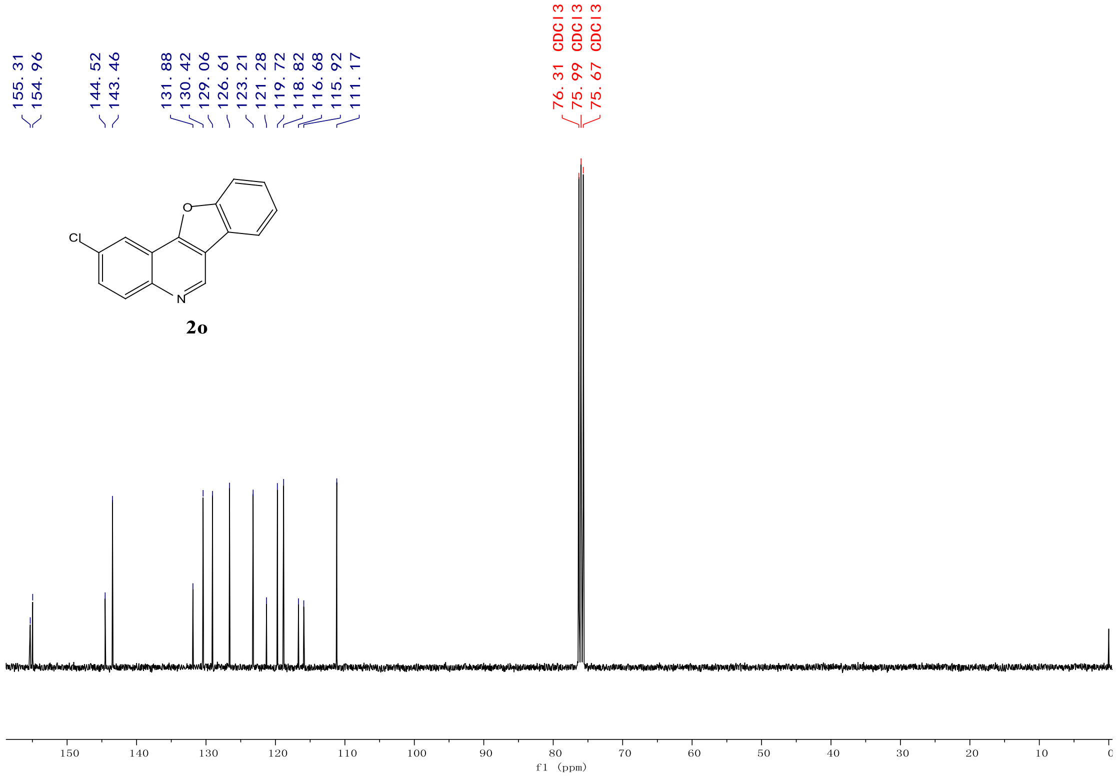
155.31
154.96
144.52
143.46
131.88
130.42
129.06
126.61
123.21
121.28
119.72
118.82
116.68
115.92
111.17
76.31 CDCl3
75.99 CDCl3
75.67 CDCl3
Cl
O
N
2o
f1 (ppm)

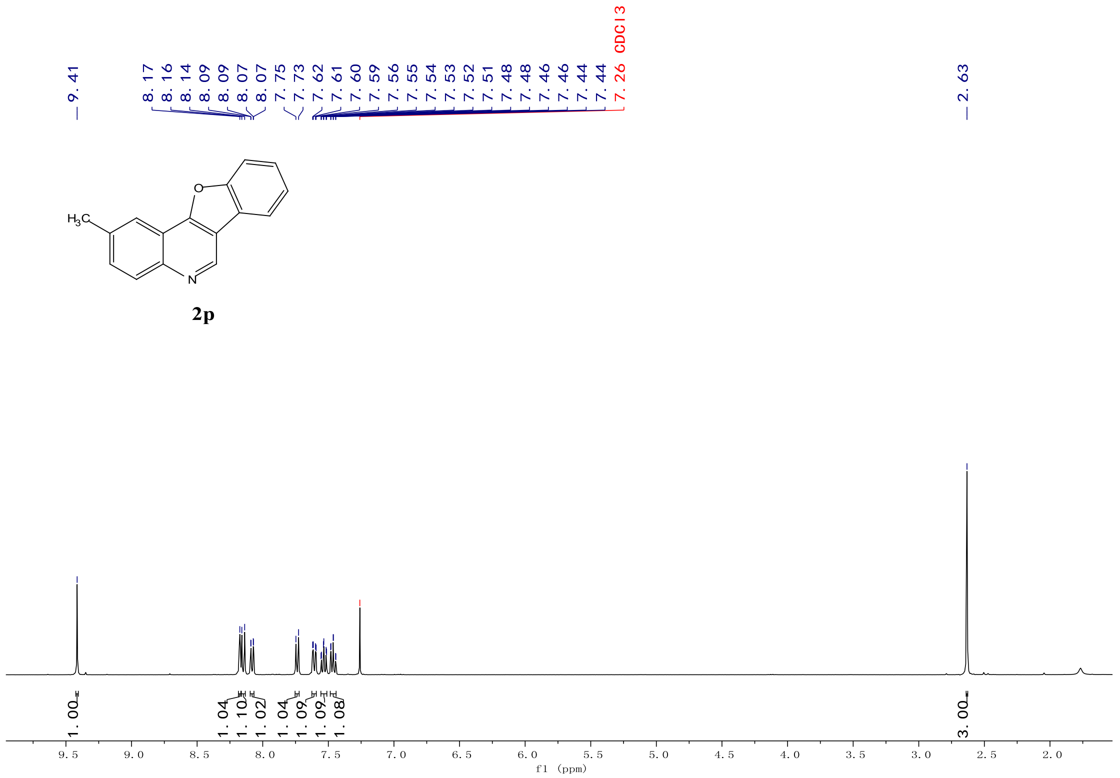
9.41
8.17
8.16
8.14
8.09
8.09
8.07
8.07
7.75
7.73
7.62
7.61
7.60
7.59
7.56
7.55
7.54
7.53
7.52
7.51
7.48
7.48
7.46
7.46
7.44
7.44
7.26 CDCl3
2.63
H3C
O
N
2p
1.00
1.04
1.10
1.02
1.04
1.09
1.09
1.08
3.00
f1 (ppm)

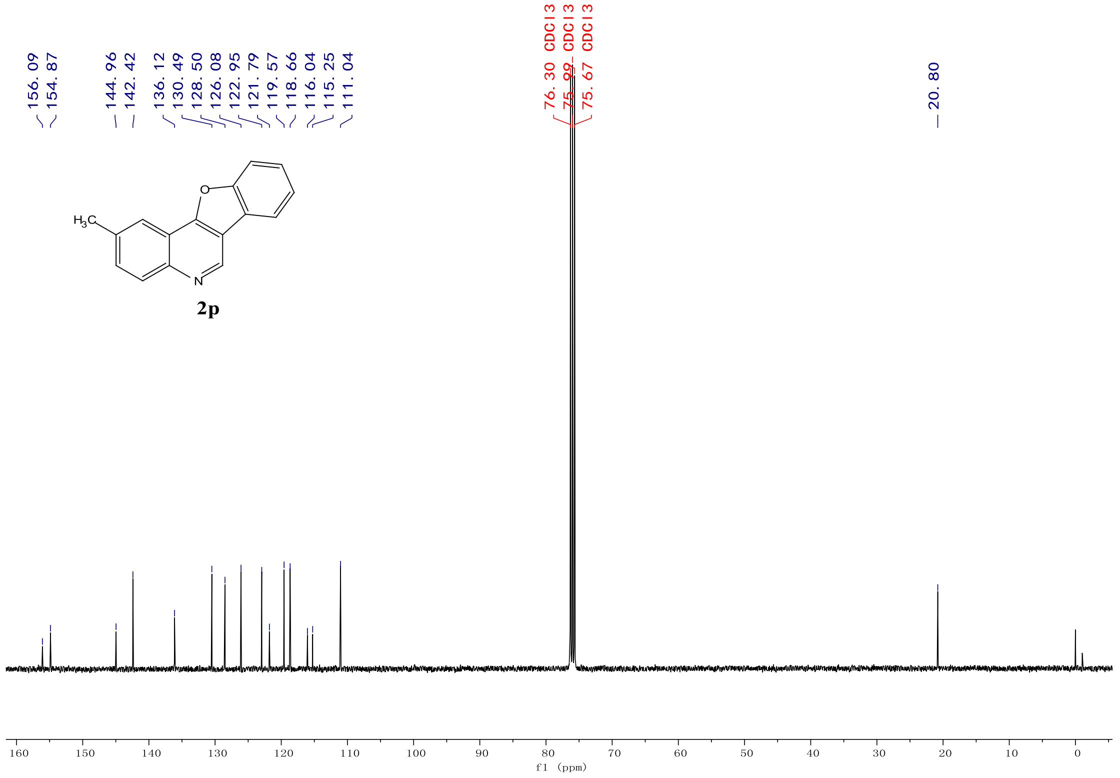
156.09
154.87
144.96
142.42
136.12
130.49
128.50
126.08
122.95
121.79
119.57
118.66
116.04
115.25
111.04
76.30 CDCl3
75.99 CDCl3
75.67 CDCl3
20.80
H3C
O
N
2p
f1 (ppm)

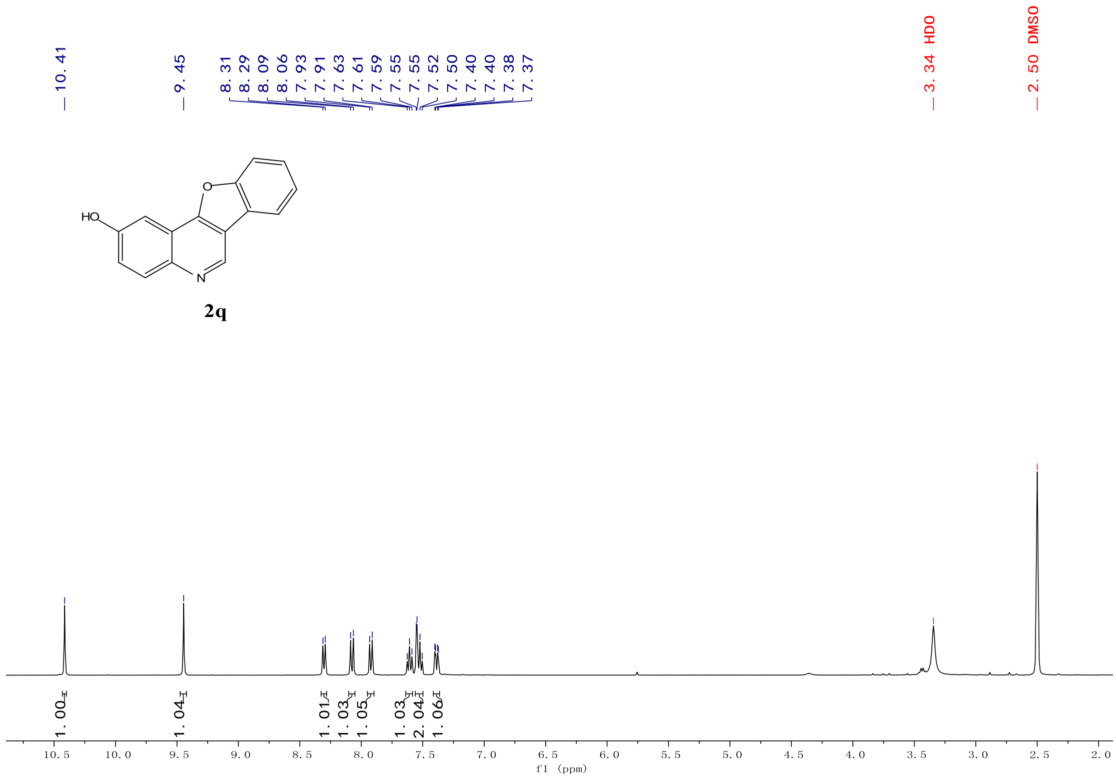
10.41
9.45
8.31 8.29 8.09 8.06 7.93 7.91 7.63 7.61 7.59 7.55 7.55 7.52 7.50 7.40 7.38 7.37
3.34 HDO
2.50 DMSO
HO
O
N
2q
1.00
1.04
1.01
1.03
1.05
1.03
2.04
1.06
10.5 10.0 9.5 9.0 8.5 8.0 7.5 7.0 6.5 6.0 5.5 5.0 4.5 4.0 3.5 3.0 2.5 2.0
f1 (ppm)

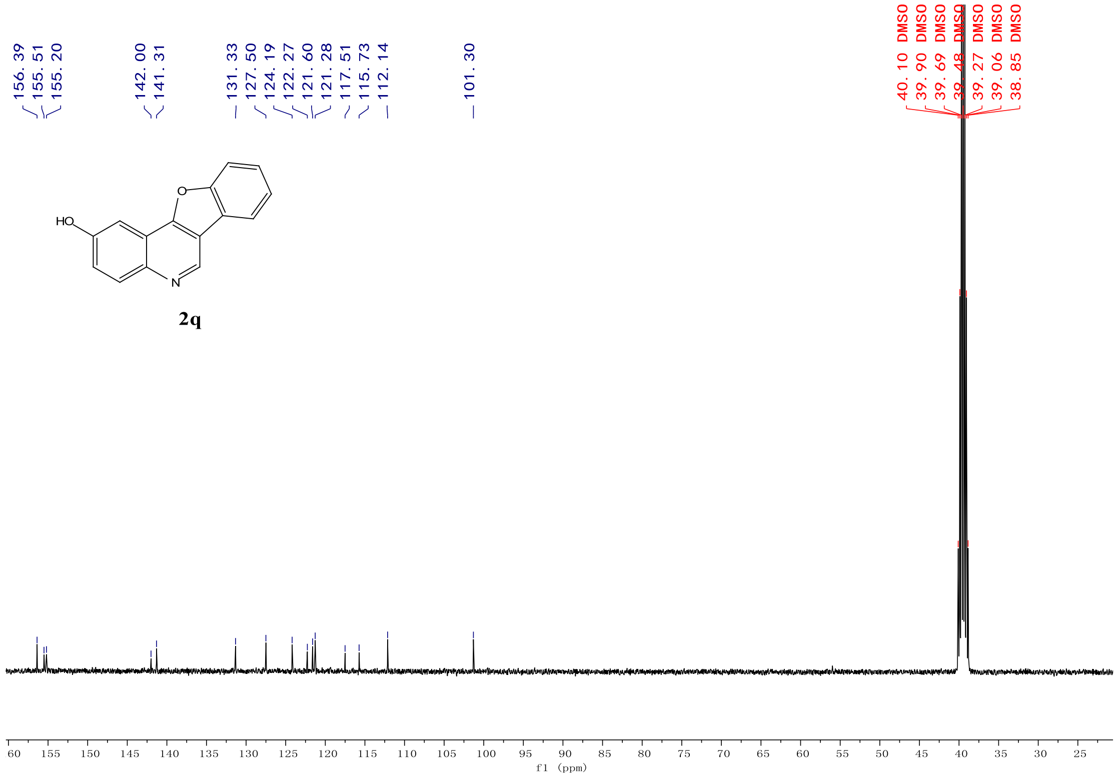
156.39 155.51 155.20
142.00 141.31
131.33 127.50 124.19 122.27 121.60 121.28 117.51 115.73 112.14
101.30
40.10 DMSO 39.90 DMSO 39.69 DMSO 39.48 DMSO 39.27 DMSO 39.06 DMSO 38.85 DMSO
HO
O
N
2q
160 155 150 145 140 135 130 125 120 115 110 105 100 95 90 85 80 75 70 65 60 55 50 45 40 35 30 25
f1 (ppm)

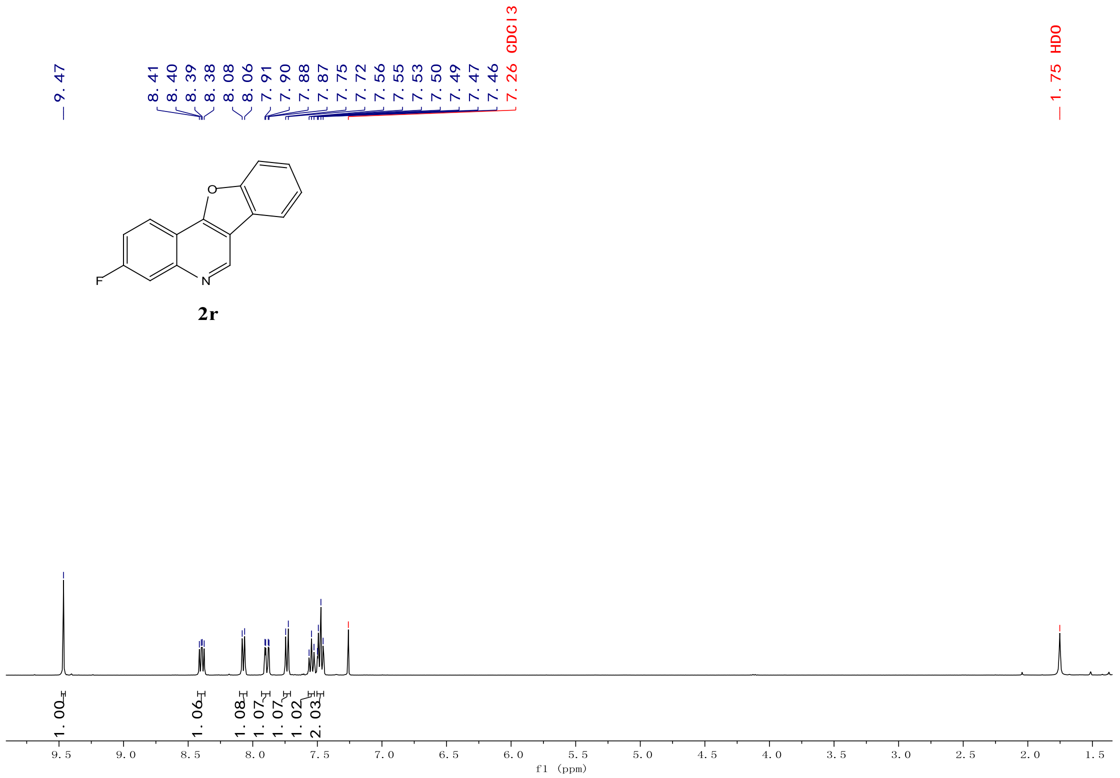
9.47
8.41
8.40
8.39
8.38
8.08
8.06
7.91
7.90
7.88
7.87
7.75
7.72
7.56
7.55
7.53
7.50
7.49
7.47
7.46
7.26 CDCl3
1.75 HDO
2r
1.00
1.06
1.08
1.07
1.07
1.02
2.03
f1 (ppm)

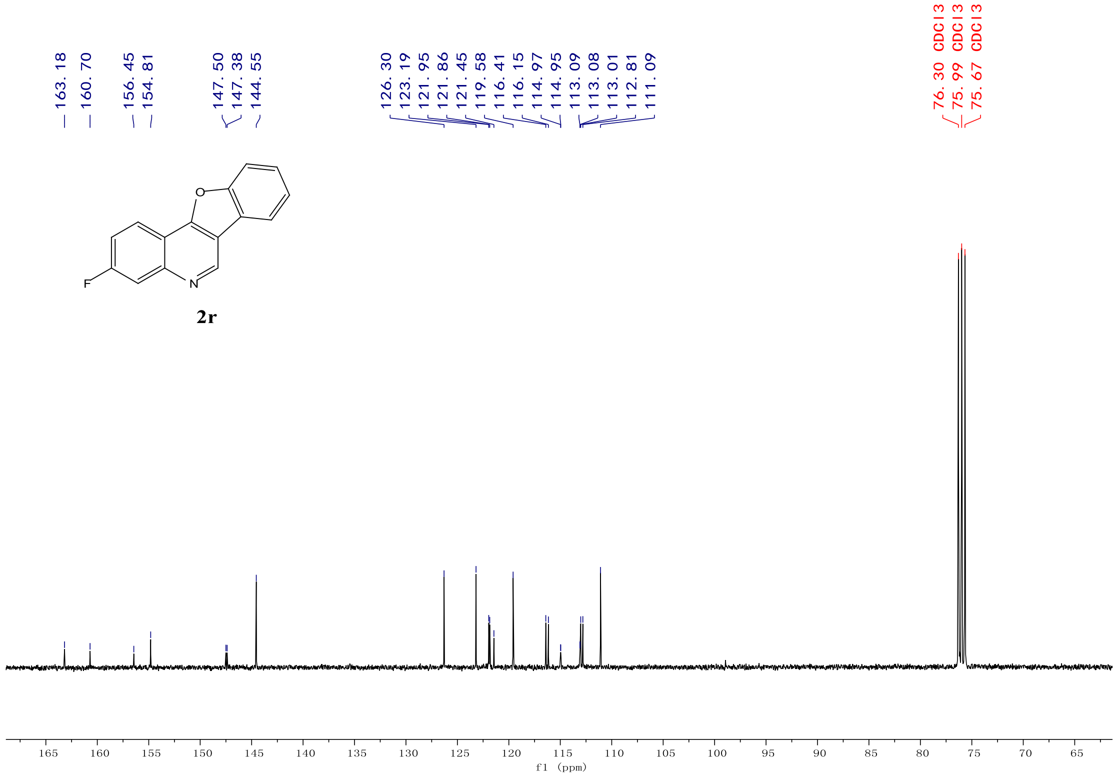
163.18
160.70
156.45
154.81
147.50
147.38
144.55
126.30
123.19
121.95
121.86
121.45
119.58
116.41
116.15
114.97
114.95
113.09
113.08
113.01
112.81
111.09
76.30 CDCl3
75.99 CDCl3
75.67 CDCl3
2r
f1 (ppm)

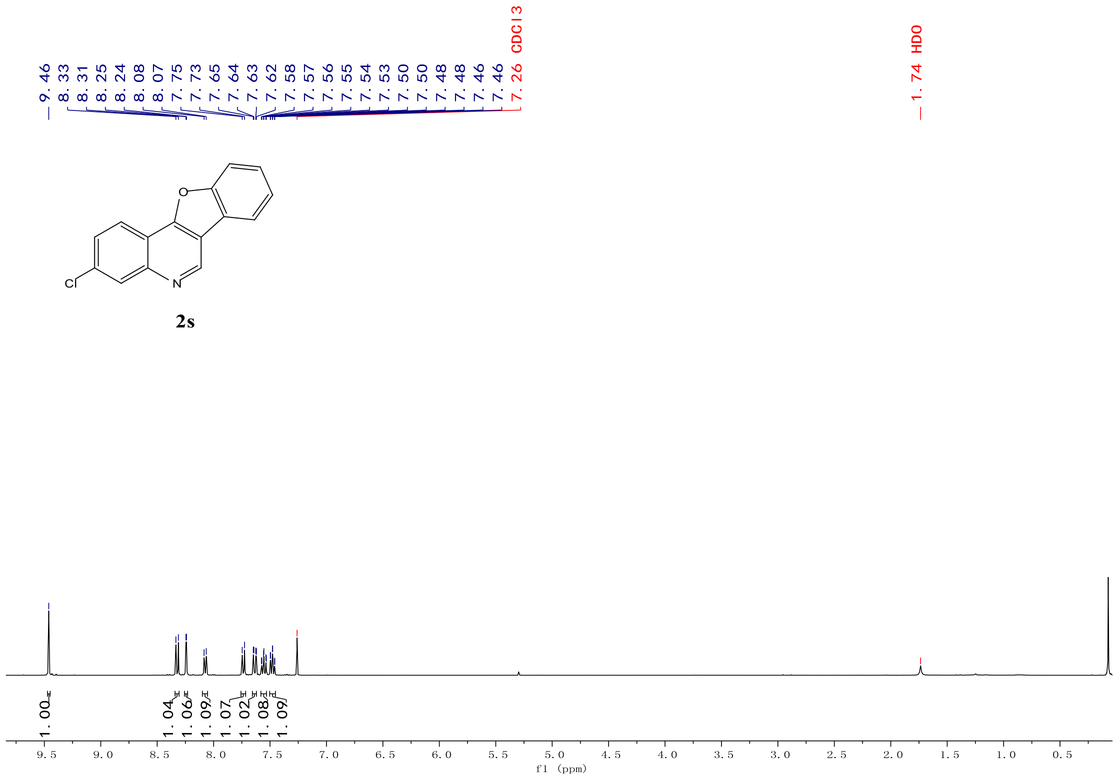
9.46
8.33
8.31
8.25
8.24
8.08
8.07
7.75
7.73
7.65
7.64
7.63
7.62
7.58
7.57
7.56
7.55
7.54
7.53
7.50
7.50
7.48
7.48
7.46
7.46
7.26 CDCl3
1.74 HDO
O
Cl
N
2s
1.00
1.04
1.06
1.09
1.07
1.02
1.08
1.09
9.5 9.0 8.5 8.0 7.5 7.0 6.5 6.0 5.5 5.0 4.5 4.0 3.5 3.0 2.5 2.0 1.5 1.0 0.5
f1 (ppm)

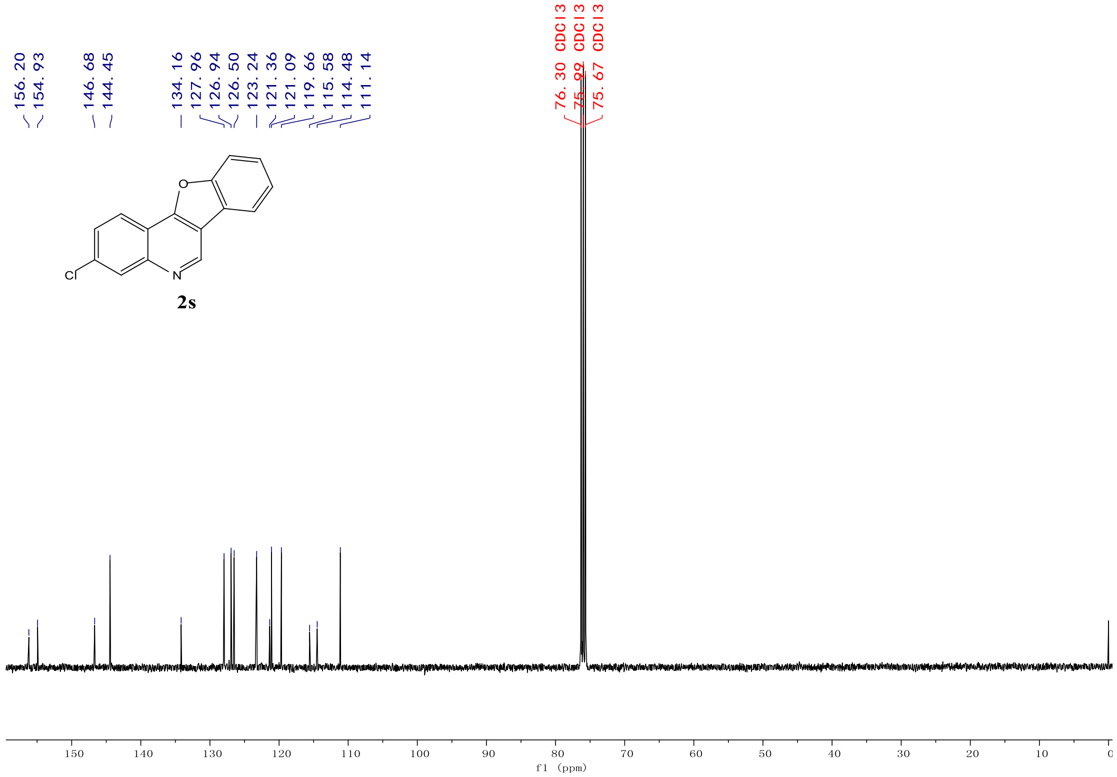
156.20
154.93
146.68
144.45
134.16
127.96
126.94
126.50
123.24
121.36
121.09
119.66
115.58
114.48
111.14
76.30 CDCl3
CDCl3
75.67 CDCl3
O
Cl
N
2s
150 140 130 120 110 100 90 80 70 60 50 40 30 20 10 0
f1 (ppm)

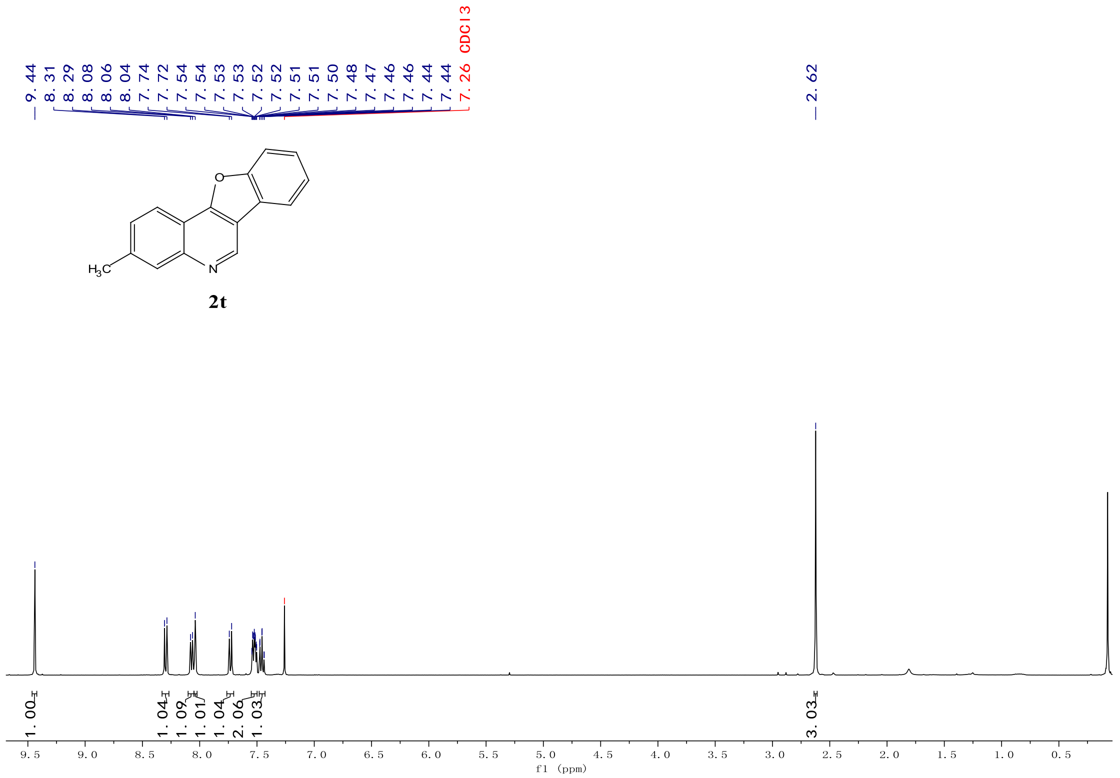
9.44 8.31 8.29 8.08 8.06 8.04 7.74 7.72 7.54 7.54 7.53 7.53 7.52 7.52 7.51 7.51 7.50 7.48 7.47 7.46 7.44 7.44
7.26 CDCl3
2.62
H_3C
N
O
2t
1.00 1.04 1.09 1.01 1.04 2.06 1.03 3.03
9.5 9.0 8.5 8.0 7.5 7.0 6.5 6.0 5.5 5.0 4.5 4.0 3.5 3.0 2.5 2.0 1.5 1.0 0.5
f1 (ppm)

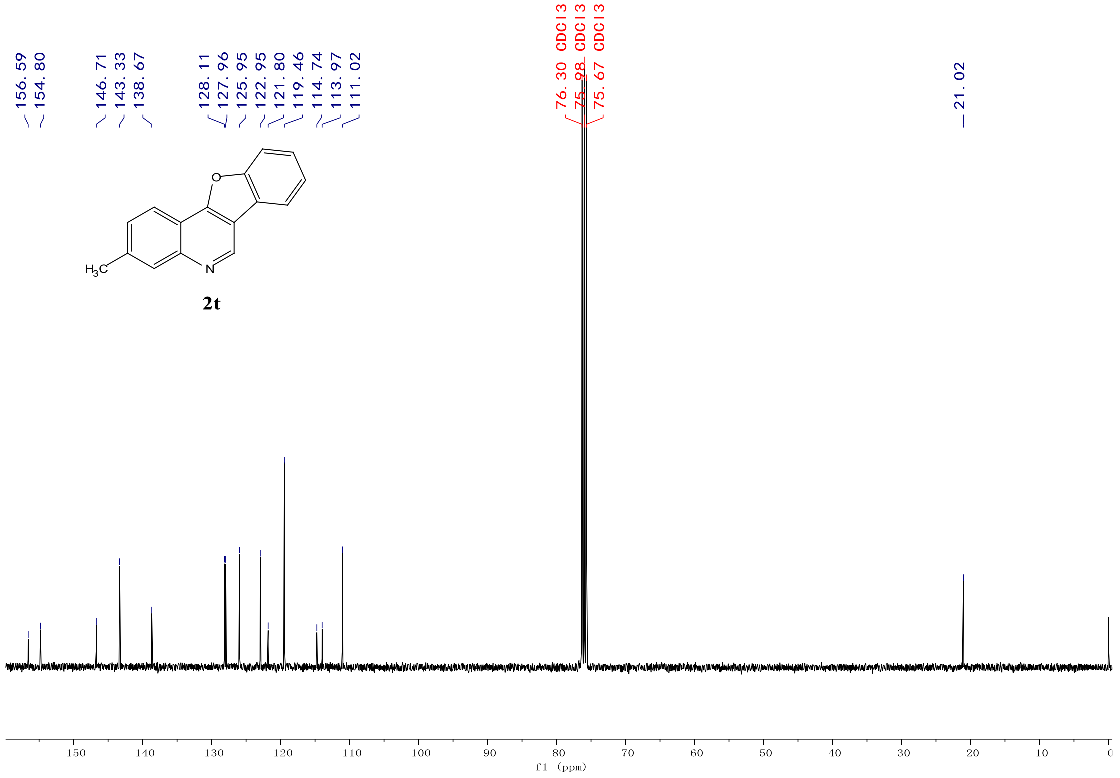
156.59 154.80 146.71 143.33 138.67 128.11 127.96 125.95 122.95 121.80 119.46 114.74 113.97 111.02
76.30 CDCl3
75.98 CDCl3
75.67 CDCl3
21.02
H_3C
N
O
2t
150 140 130 120 110 100 90 80 70 60 50 40 30 20 10 0
f1 (ppm)

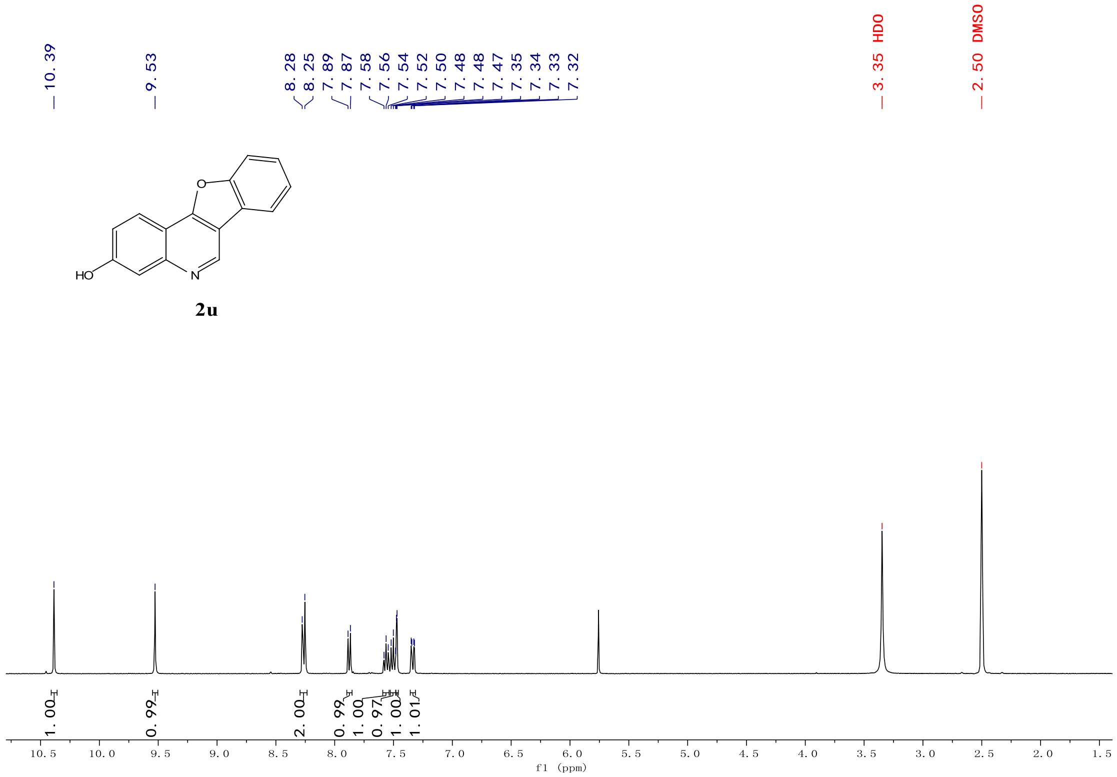
10.39
9.53
8.28
8.25
7.89
7.87
7.58
7.56
7.54
7.52
7.50
7.48
7.48
7.47
7.35
7.34
7.33
7.32
3.35 HDO
2.50 DMSO
HO
N
O
2u
1.00
0.99
2.00
0.99
1.00
0.97
1.00
1.01
10.5 10.0 9.5 9.0 8.5 8.0 7.5 7.0 6.5 6.0 5.5 5.0 4.5 4.0 3.5 3.0 2.5 2.0 1.5
f1 (ppm)

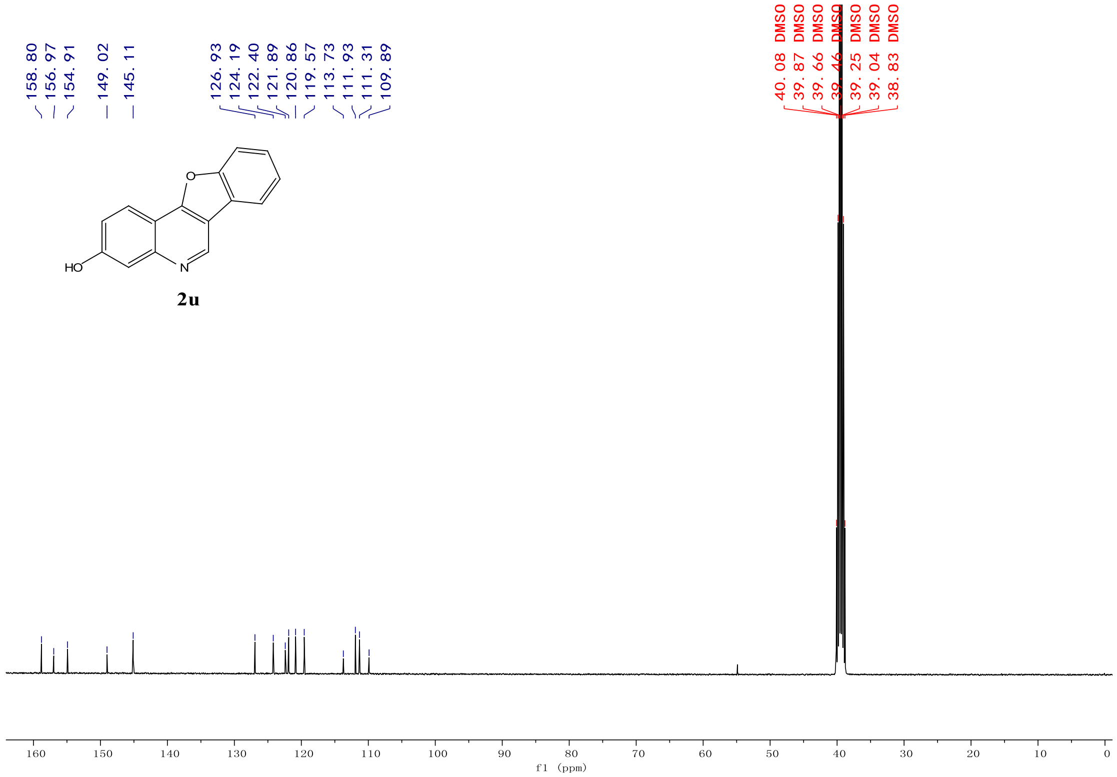
158.80
156.97
154.91
149.02
145.11
126.93
124.19
122.40
121.89
120.86
119.57
113.73
111.93
111.31
109.89
40.08 DMSO
39.87 DMSO
39.66 DMSO
39.46 DMSO
39.25 DMSO
39.04 DMSO
38.83 DMSO
HO
N
O
2u
160 150 140 130 120 110 100 90 80 70 60 50 40 30 20 10 0
f1 (ppm)

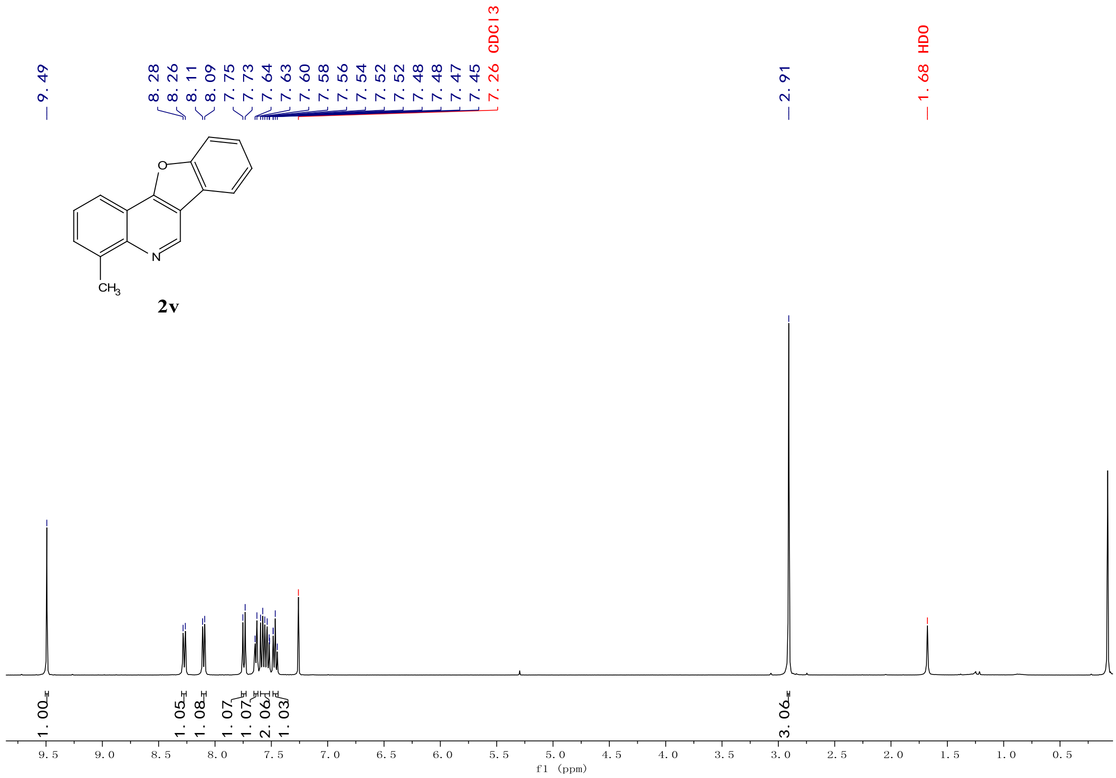
9.49
8.28
8.26
8.11
8.09
7.75
7.73
7.64
7.63
7.60
7.58
7.56
7.54
7.52
7.52
7.48
7.48
7.47
7.45
7.26 CDCl3
2.91
1.68 HDO
1.00
1.05
1.08
1.07
1.07
2.06
1.03
3.06
f1 (ppm)

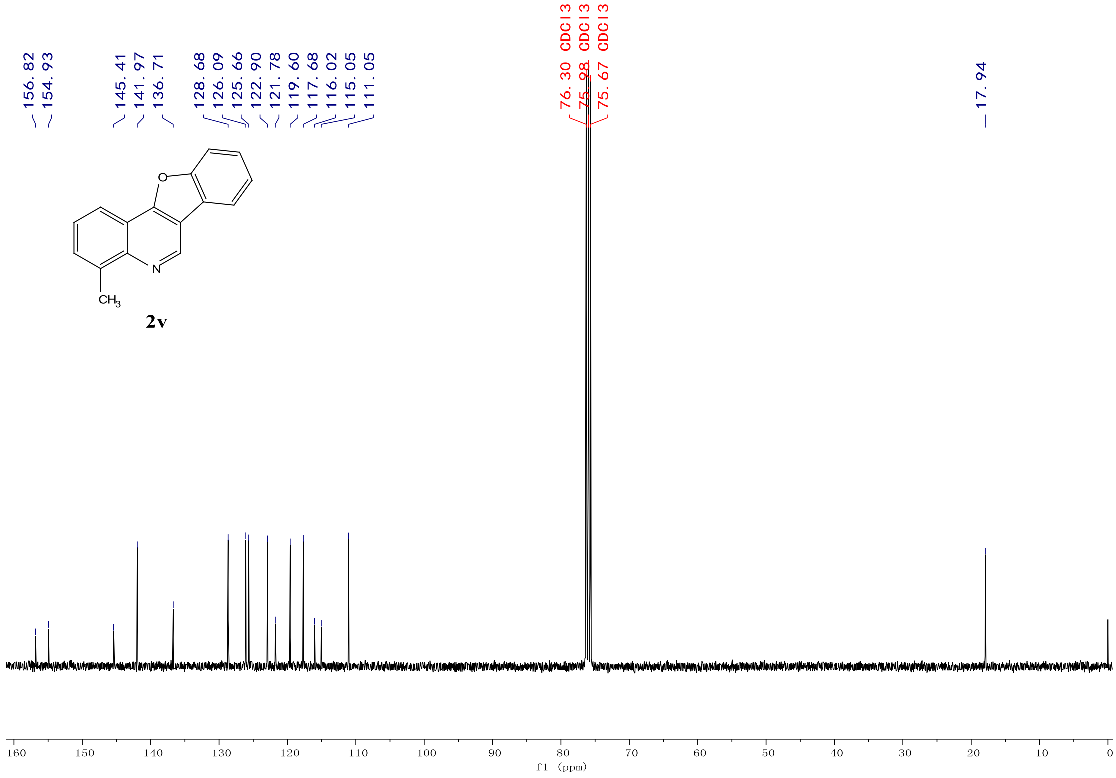
156.82
154.93
145.41
141.97
136.71
128.68
126.09
125.66
122.90
121.78
119.60
117.68
116.02
115.05
111.05
76.30 CDCl3
75.98 CDCl3
75.67 CDCl3
17.94
f1 (ppm)

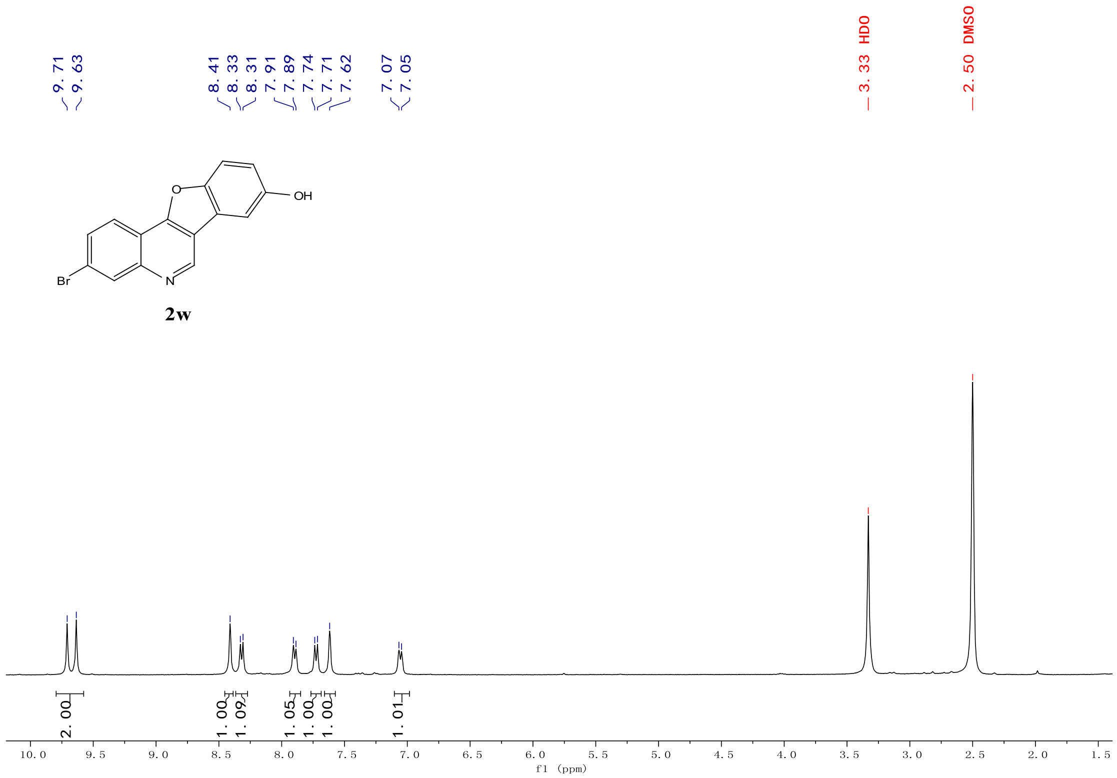

9.71
9.63
8.41
8.33
8.31
7.91
7.89
7.74
7.71
7.62
7.07
7.05
3.33 HDO
2.50 DMSO
OH
O
Br
N
2w
2.00
1.00
1.09
1.05
1.00
1.00
1.01
f1 (ppm)

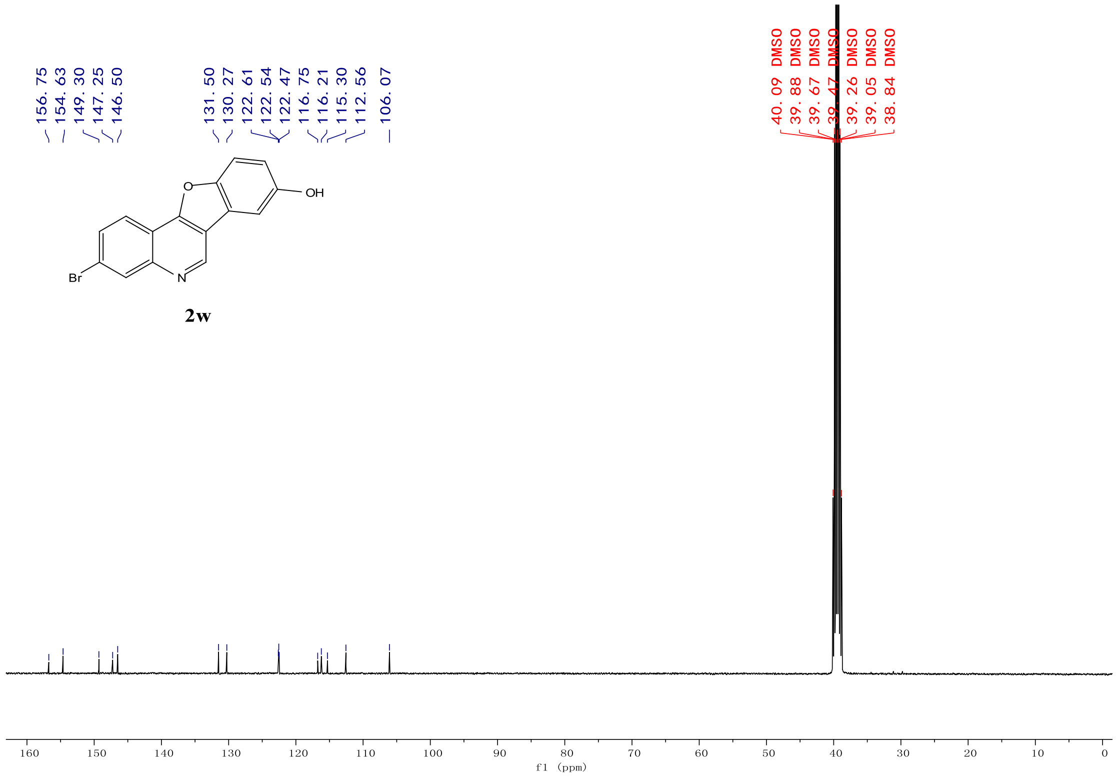

156.75
154.63
149.30
147.25
146.50
131.50
130.27
122.61
122.54
122.47
116.75
116.21
115.30
112.56
106.07
40.09 DMSO
39.88 DMSO
39.67 DMSO
39.47 DMSO
39.26 DMSO
39.05 DMSO
38.84 DMSO
OH
O
Br
N
2w
f1 (ppm)

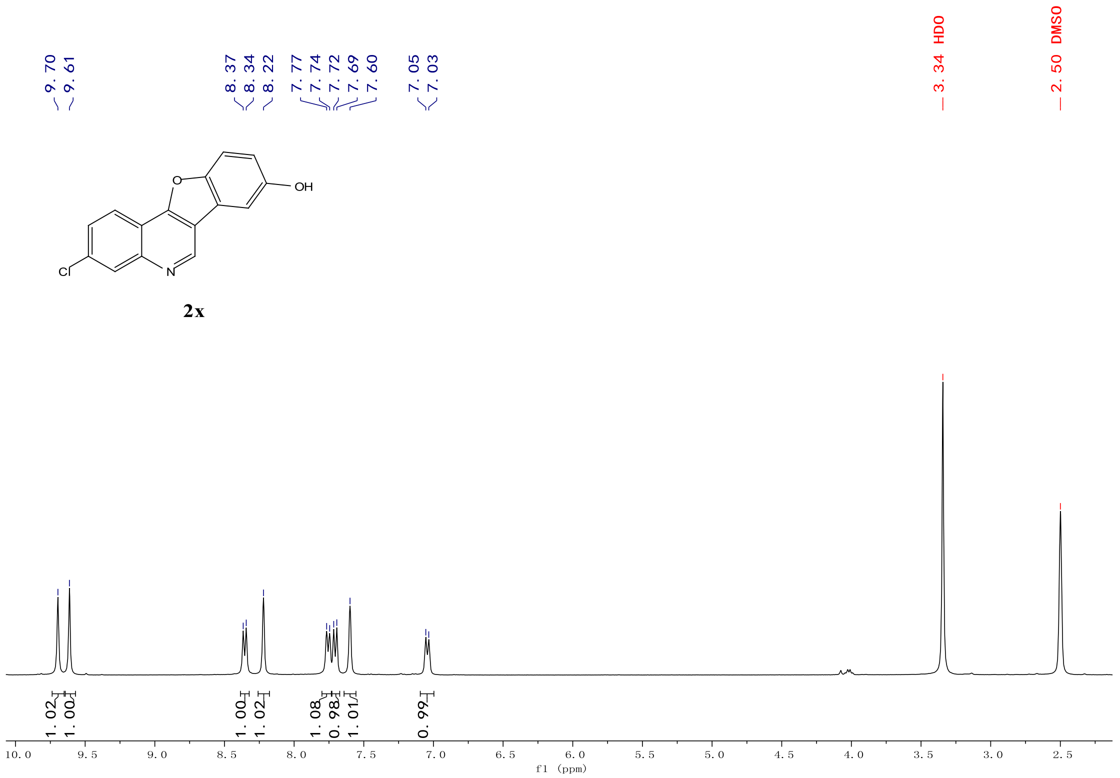
9.70
9.61
8.37
8.34
8.22
7.77
7.74
7.72
7.69
7.60
7.05
7.03
3.34 HDO
2.50 DMSO
OH
O
N
Cl
2x
1.02
1.00
1.00
1.02
1.08
0.98
1.01
0.99
f1 (ppm)

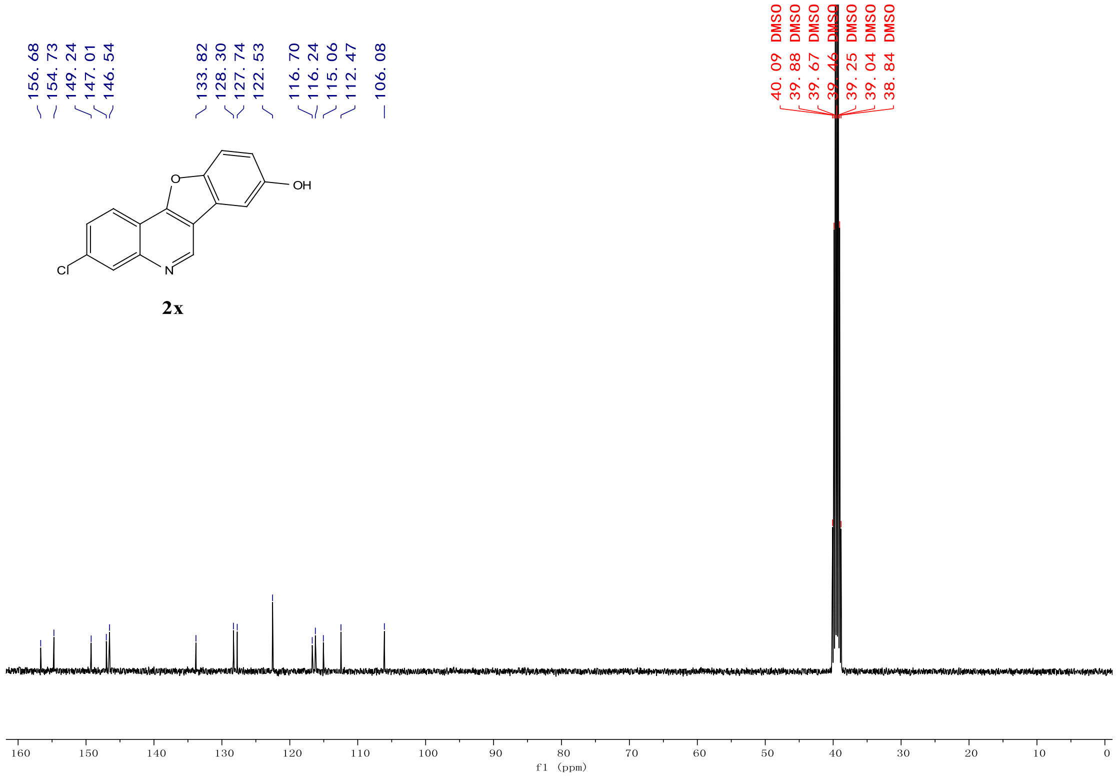
156.68
154.73
149.24
147.01
146.54
133.82
128.30
127.74
122.53
116.70
116.24
115.06
112.47
106.08
40.09 DMSO
39.88 DMSO
39.67 DMSO
39.46 DMSO
39.25 DMSO
39.04 DMSO
38.84 DMSO
OH
O
N
Cl
2x
f1 (ppm)
